# Supplementary material for: Translational reprogramming in response to accumulating stressors ensures critical threshold levels of Hsp90 for mammalian life
Source: Nat Commun. 2022 Oct 21;13:6271. doi: 10.1038/s41467-022-33916-3 (PMC9587034; doi:10.1038/s41467-022-33916-3)
Supplement: Supplementary file 1 — Supplementary Information [file 41467_2022_33916_MOESM1_ESM.pdf]

Supplementary Information

Translational reprogramming in response to accumulating stressors ensures critical threshold levels of Hsp90 for mammalian life

Bhattacharya et al.

Supplementary Information includes 13 Supplementary Figures with corresponding legends, a Supplementary Table, and Supplementary References.

Supplementary Figure 1

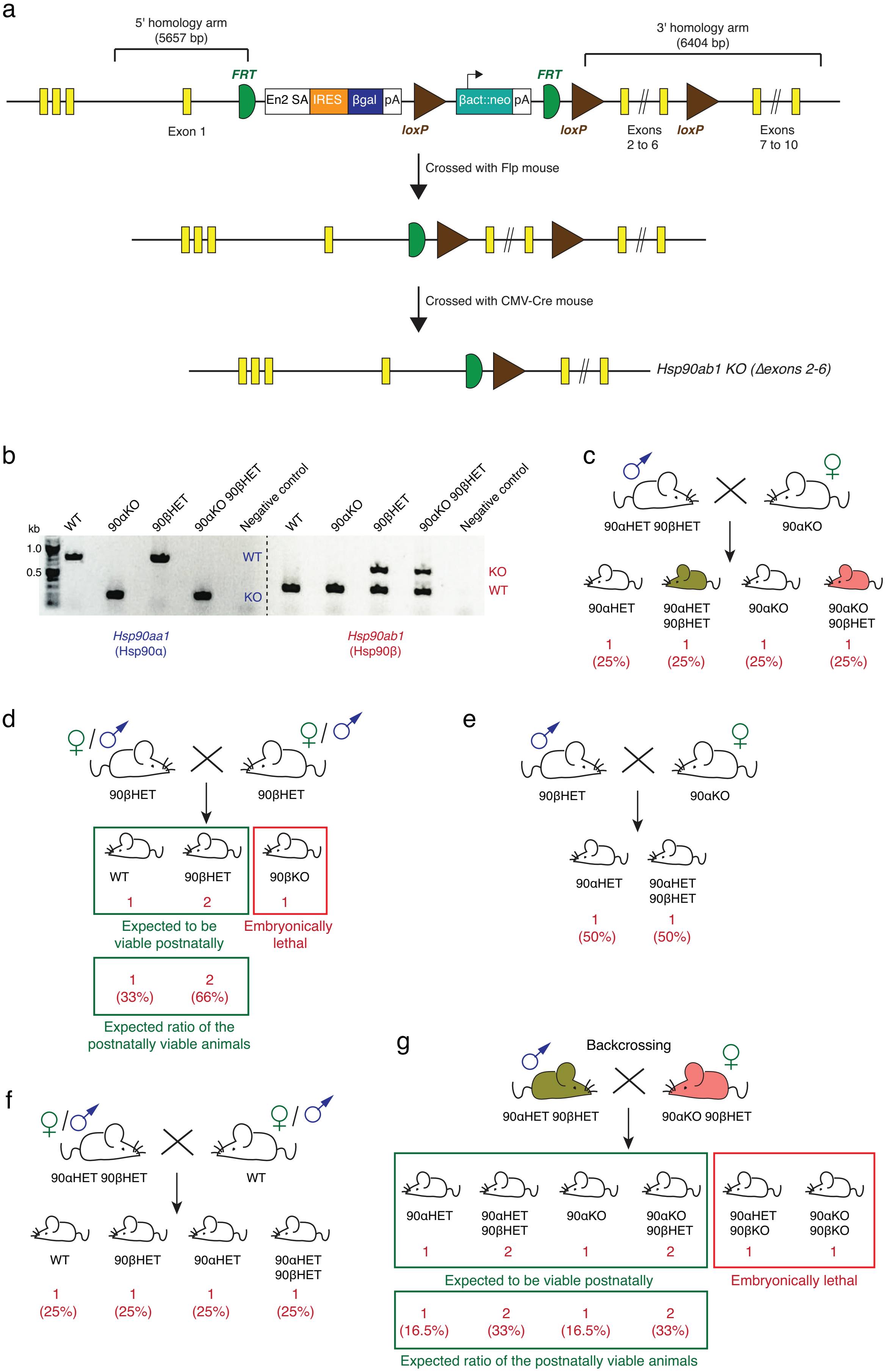

Supplementary Fig. 1 Breeding schemes and genotyping. a, Schematic representation of targeted allele of mouse *Hsp90ab1* and its stepwise deletion to create the *Hsp90ab1* KO allele (see Methods for details). **b,** A representative profile of PCR-mediated mouse genotyping. **c-g,** Schemes of different mouse breeding strategies used in this study. Genotypes of offspring and their expected ratios were calculated based on Mendelian inheritance.

Supplementary Figure 2

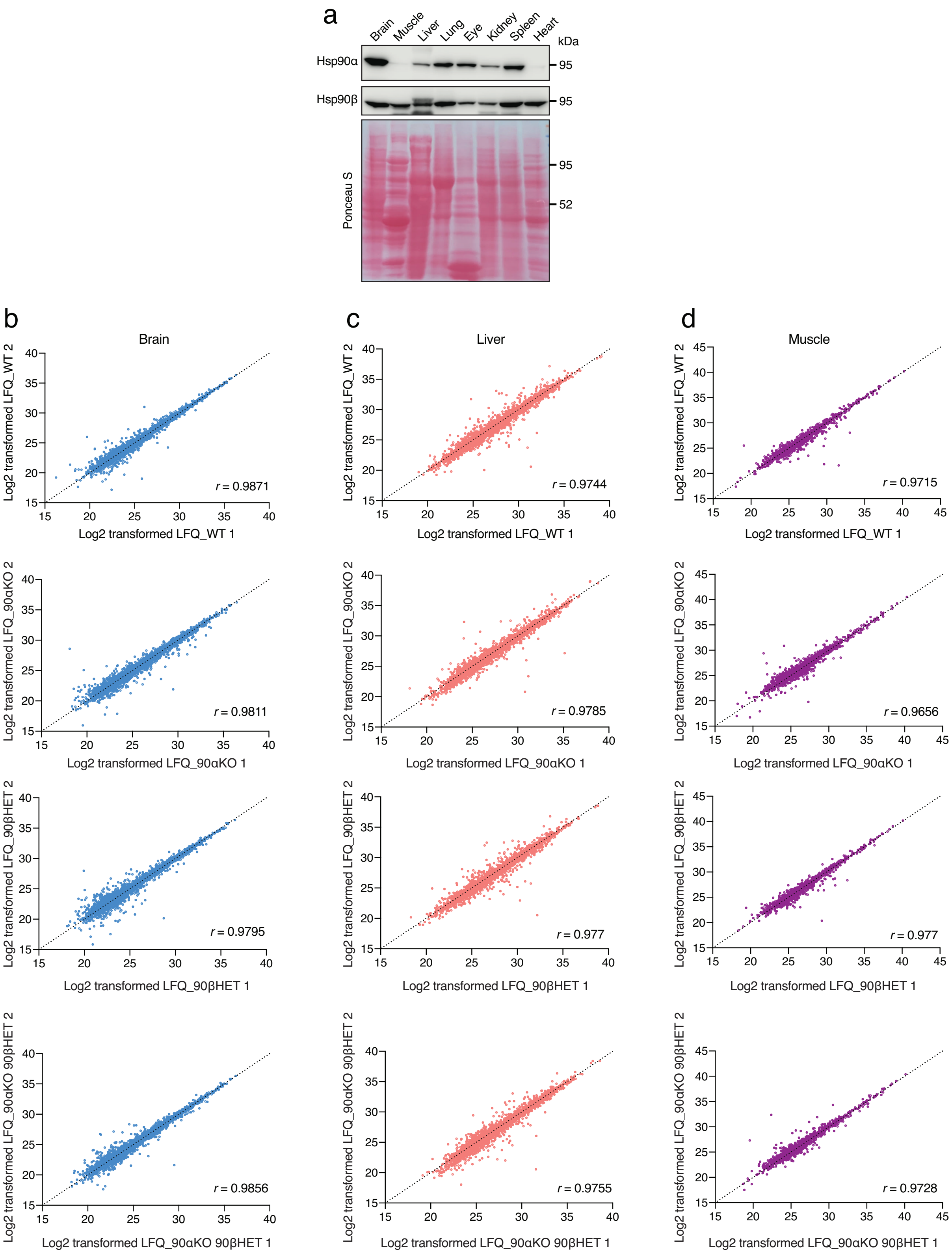

Supplementary Fig. 2 Quality control of proteomic datasets. a, Immunoblots of Hsp90α and Hsp90β from the indicated WT mouse tissues (this initial quality control experiment was done with only one set of WT mouse tissues). The Ponceau S-stained nitrocellulose filters serve as the loading control. **b-d,** Scatter plots and correlation

analyses between two biological replicates (n = 2 biologically independent samples) of the indicated genotypes and tissues. Log2 transformed LFQ values were plotted. The comparisons between samples from different tissues are shown in different colors. *r*, Pearson correlation coefficient. Source data are provided as a Source Data file.

Supplementary Figure 3

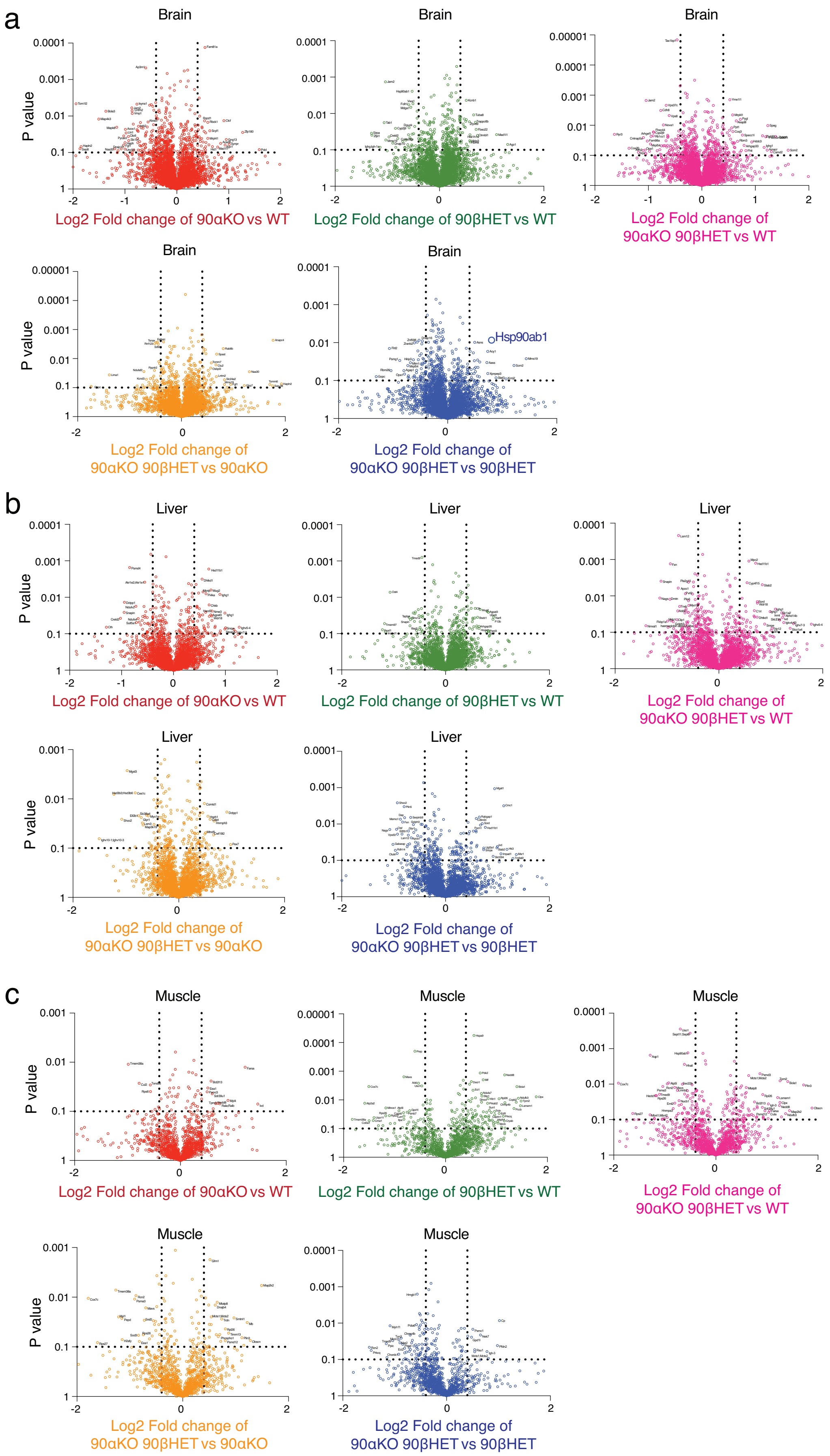

Supplementary Fig. 3 Negligible proteome changes in Hsp90 mutant mouse tissues. a-c, Volcano plots of the normalized fold changes of the whole tissue proteomes of WT and Hsp90 mutant mice. Log2 fold change of > 0.4 or < -0.4 with a p-value of < 0.1 were considered significant differences for a particular protein. The comparisons between samples of different genotypes are shown in different colors (n = 2 biologically independent samples). The statistical significance between groups was analyzed by two-tailed unpaired Student's t-tests.

Supplementary Figure 4

a

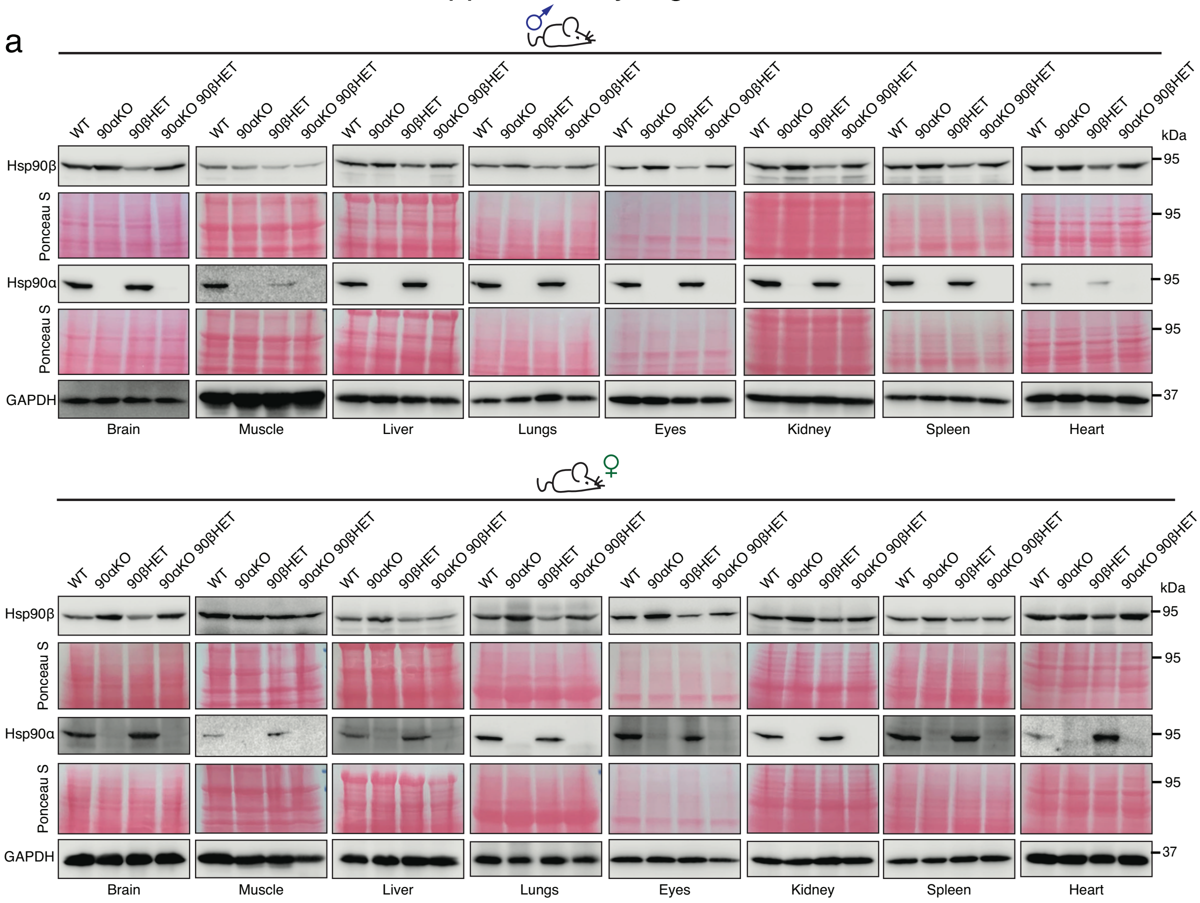

b

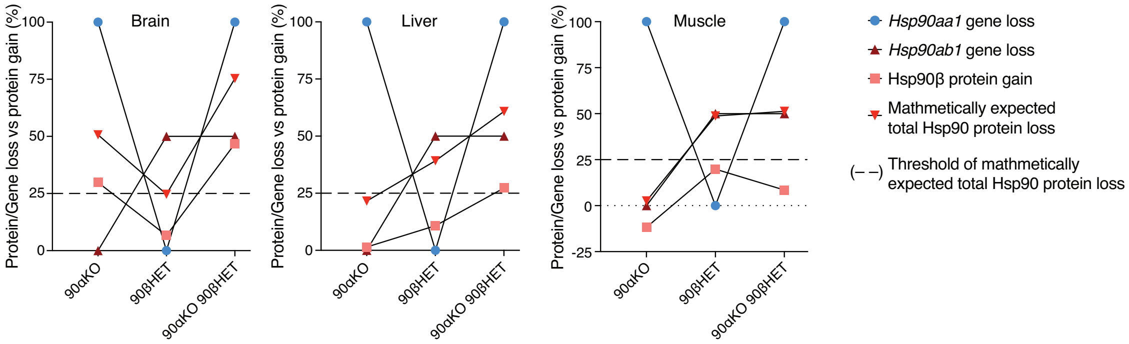

c

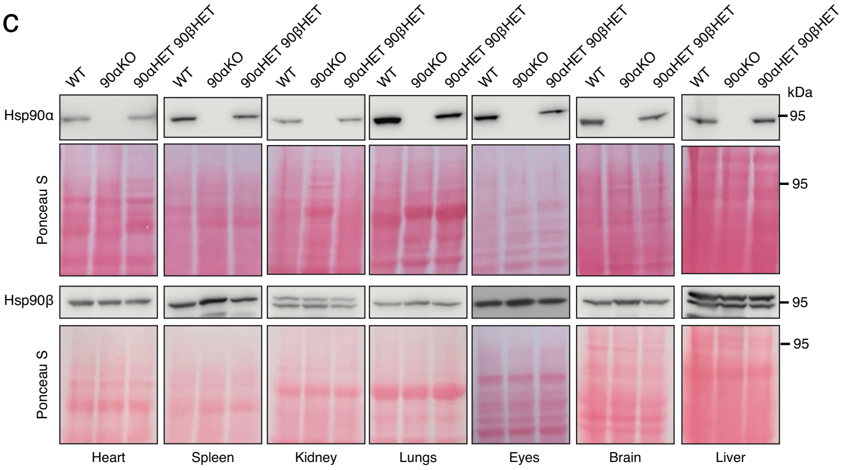

Supplementary Fig. 4 Correlation between increased Hsp90β protein levels and *Hsp90* allele-specific losses. a, Immunoblots of Hsp90α and Hsp90β from different mouse tissues of the indicated genotypes (representatives of n = 3 biologically independent mouse tissue samples). GAPDH and the Ponceau S-stained nitrocellulose filters serve as the loading control. Data for sets of male and female mice as indicated. **b,** Line graphs represent the correlation between Hsp90β protein gain and corresponding *Hsp90aa1/Hsp90ab1* allele loss in Hsp90 mutant mouse tissues compared to that of WT (n = 2 biologically independent samples). % gain of Hsp90β protein was calculated by subtracting the expected abundance from the actual abundance of Hsp90β for a particular genotype, and % mathematically expected total Hsp90 protein loss was calculated by subtracting the expected abundance of total Hsp90 from 100% (WT levels) for a particular genotype using the data of the whole tissue proteomic analyses illustrated in Fig. 2b. **c,** Immunoblots of Hsp90α and Hsp90β from different mouse tissues of the indicated genotypes (representative of n = 2 biologically independent mouse tissue samples). Source data are provided as a Source Data file.

Supplementary Figure 5

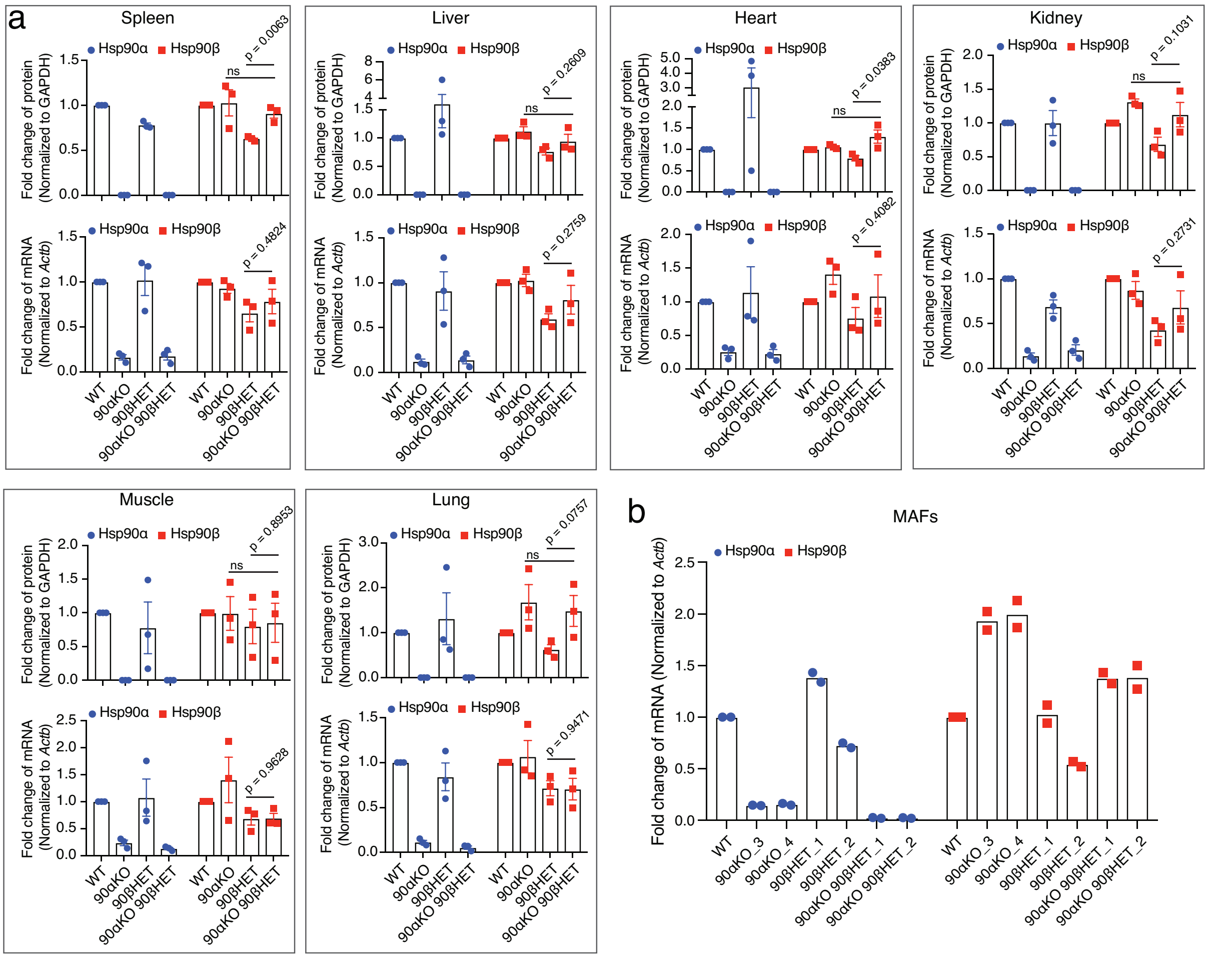

Supplementary Fig. 5 Comparisons between mRNA and protein levels of Hsp90 isoforms. a, Bar graphs represent normalized protein and mRNA expression of Hsp90α and Hsp90β isoforms in mouse tissues of the indicated genotypes. WT protein or mRNA levels were set to 1 (n = 3 biologically independent samples). **b,** mRNA expression of Hsp90α and Hsp90β in the Hsp90 mutant MAFs relative to WT MAFs (set to 1). Two independent clones of Hsp90 mutant MAFs were analyzed (n = 2 biologically independent samples). The bar graphs show the mean values ± SEM. The statistical significance between the groups was analyzed by two-tailed unpaired Student's t-tests. ns, non-significant p values. Source data are provided as a Source Data file.

Supplementary Figure 6

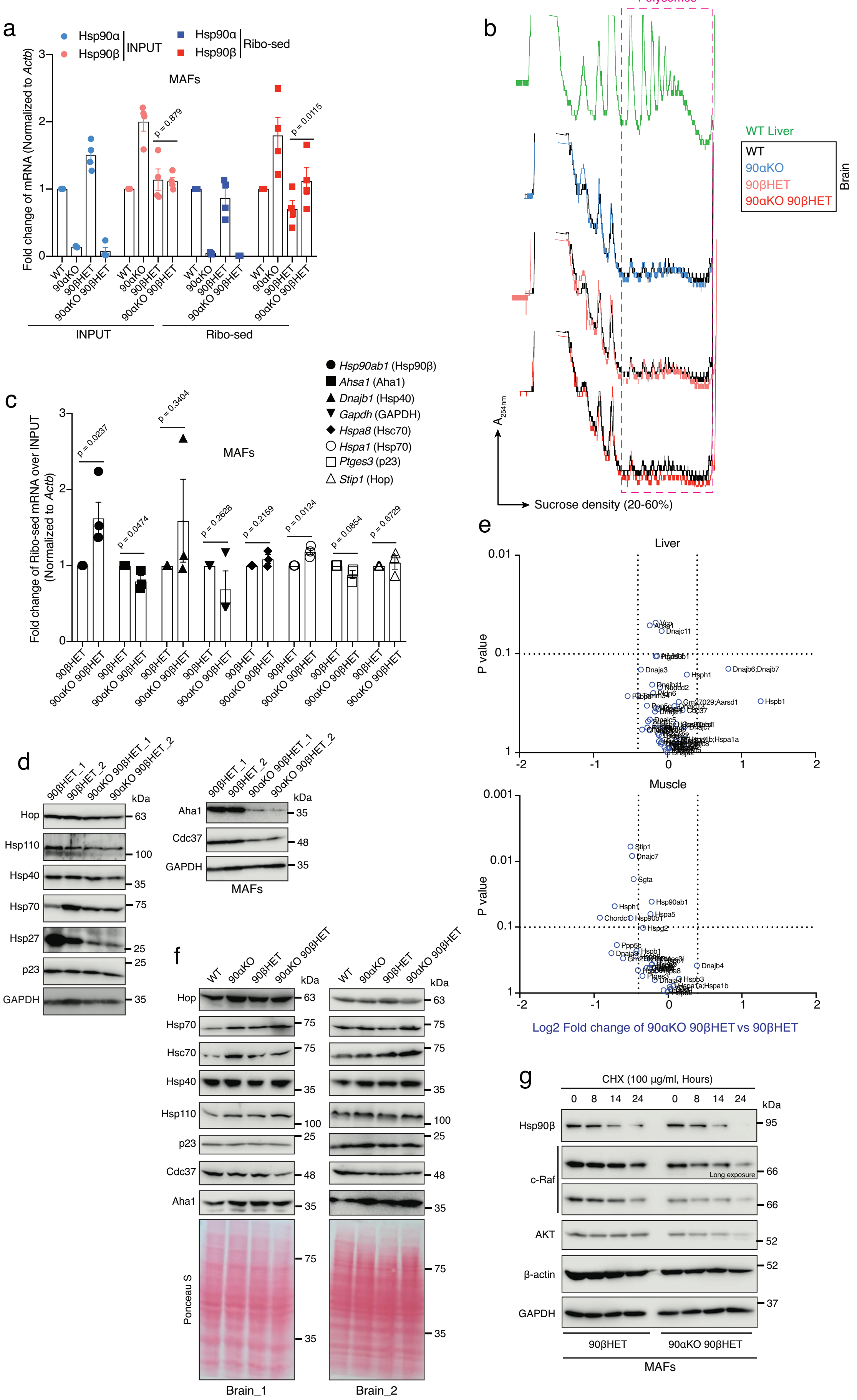

Supplementary Fig. 6 Hsp90β-specific translational reprogramming in 90αKO 90βHET cells and tissues. a, Normalized fold change of total and ribosome-associated Hsp90α and Hsp90β mRNAs in MAFs of the indicated genotypes (see also Fig. 3c; n = 4 biologically independent samples). WT mRNA levels were set to 1. *Actb* was used as a reference gene. **b,** Representative polysome profiles of mouse brain (set 2) as illustrated in Fig. 3d. The polysome profile of WT mouse liver was used to align accurately the less prominent brain polysome peaks. **c,** Normalized fold change of ribosome-associated mRNAs of different molecular chaperones and co-chaperones over inputs in 90αKO 90βHET compared to 90βHET MAFs (for *Hsp90ab1*, n = 4; for other genes, n = 3 biologically independent samples). 90βHET mRNA levels were set to 1. *Actb* was used as a reference gene. **d,** Immunoblots of different molecular chaperones and co-chaperones from two independent clones of 90βHET and 90αKO 90βHET MAFs (representative of n = 2 biologically independent experiments). GAPDH serves as the loading control. **e,** Volcano plots of the normalized fold changes of the Hsp70-Hsp90-related chaperones, co-chaperones, and other stress-responsive proteins determined by quantitative label-free proteomic analysis of liver and muscle of mice with the indicated genotypes (n = 2 biologically independent samples). Log2 fold change of > 0.4 or < -0.4 with a p-value of < 0.1 were considered significant differences. **f,** Immunoblots of different molecular chaperones and co-chaperones from brain of two independent mouse sets of the indicated genotypes. Ponceau S-stained nitrocellulose filters serve as the loading control. **g,** Cycloheximide chase assay with MAFs of the indicated genotypes to reveal protein turnover rates (representative of n = 2 biologically independent experiments). The bar graphs show the mean values ± SEM. The statistical significance between the groups was analyzed by two-tailed unpaired Student's t-tests. Source data are provided as a Source Data file.

Supplementary Figure 7

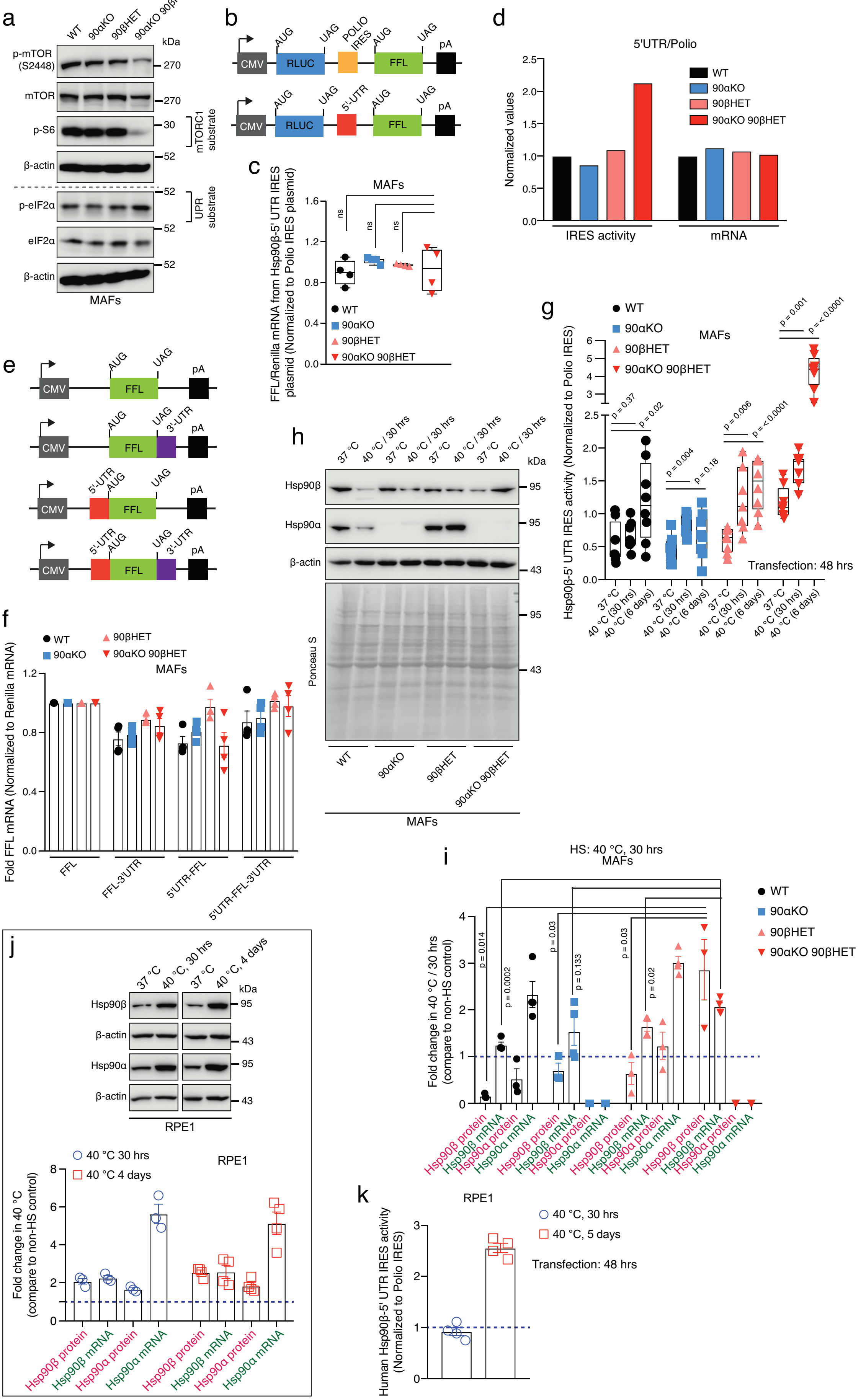

Supplementary Fig. 7 Stress-induced IRES function of the 5´-UTR of the Hsp90β mRNA. a, Immunoblots of total and phosphorylated mTOR, S6, and eIF2α from MAFs (representative of n = 3 biologically independent experiments). β-actin serves as the loading control. UPR, unfolded protein response. **b,** Schematic representation of the gene expression modules of the bicistronic reporter plasmids. RLUC, Renilla luciferase; FFL, firefly luciferase. **c**, Normalized mRNA expression of firefly luciferase from the bicistronic reporter plasmids (n = 4 biologically independent samples); ns, not statistically significant. **d,** Schematized view of the normalized average values of IRES activity and corresponding mRNA expression calculated from Fig. 3e and Supplementary Fig. 7c, respectively. WT values are set to 1. **e,** Schematic representation of the gene expression modules of the translational reporter plasmids. **f**, Impact of the UTRs of mouse Hsp90β mRNA on the corresponding mRNA abundance (n = 4 biologically independent samples). **g,** IRES activity of the 5´-UTR of mouse Hsp90β mRNA normalized to that of the poliovirus IRES under different heat-stressed conditions (30 hrs at 40 °C, n = 7 biologically independent samples; 6 days at 40 °C, n = 8 biologically independent samples). Note that IRES activities at 37 °C (n = 8 biologically independent experiments) are the same as those shown in Fig. 3e. **h,** Representative immunoblots of Hsp90α and Hsp90β from MAFs maintained at 37 °C and 40 °C for 30 hrs (representative of n = 3 biologically independent experiments). β-actin and the Ponceau S-stained nitrocellulose membrane serve as loading controls. **i,** Normalized fold change of mRNA (green labels, n = 4 biologically independent samples) and protein (pink labels, n = 3 biologically independent samples) levels of Hsp90α and Hsp90β from MAFs maintained at 40 °C for 30 hrs. Expression levels at 37 °C were set to 1 (dashed line). **j,** Top: Representative immunoblots of Hsp90α and Hsp90β from RPE1 cells maintained at 37 °C or 40 °C for 30 hrs or 4 days. β-actin serves as loading control. Bottom: Normalized fold change of mRNA (green labels) and protein (pink labels) levels of Hsp90α and Hsp90β from RPE1 cells maintained at 40 °C for 30 hrs (n = 3 biologically independent experiments) or 4 days (n = 4 biologically independent experiments). Expression levels at 37 °C were set to 1 (dashed line). **k,** IRES activity of the 5´-UTR of the human Hsp90β mRNA normalized to that of the poliovirus IRES under different heat-stressed conditions in RPE1 human cells (n = 4 biologically independent samples). The bar graphs show the mean values ± SEM. Box plots with whiskers show the data distribution from minima to maxima, and the lines across the boxes indicate the median values. The statistical significance between the groups was analyzed by two-tailed unpaired Student's t-tests. Source data are provided as a Source Data file.

Supplementary Figure 8

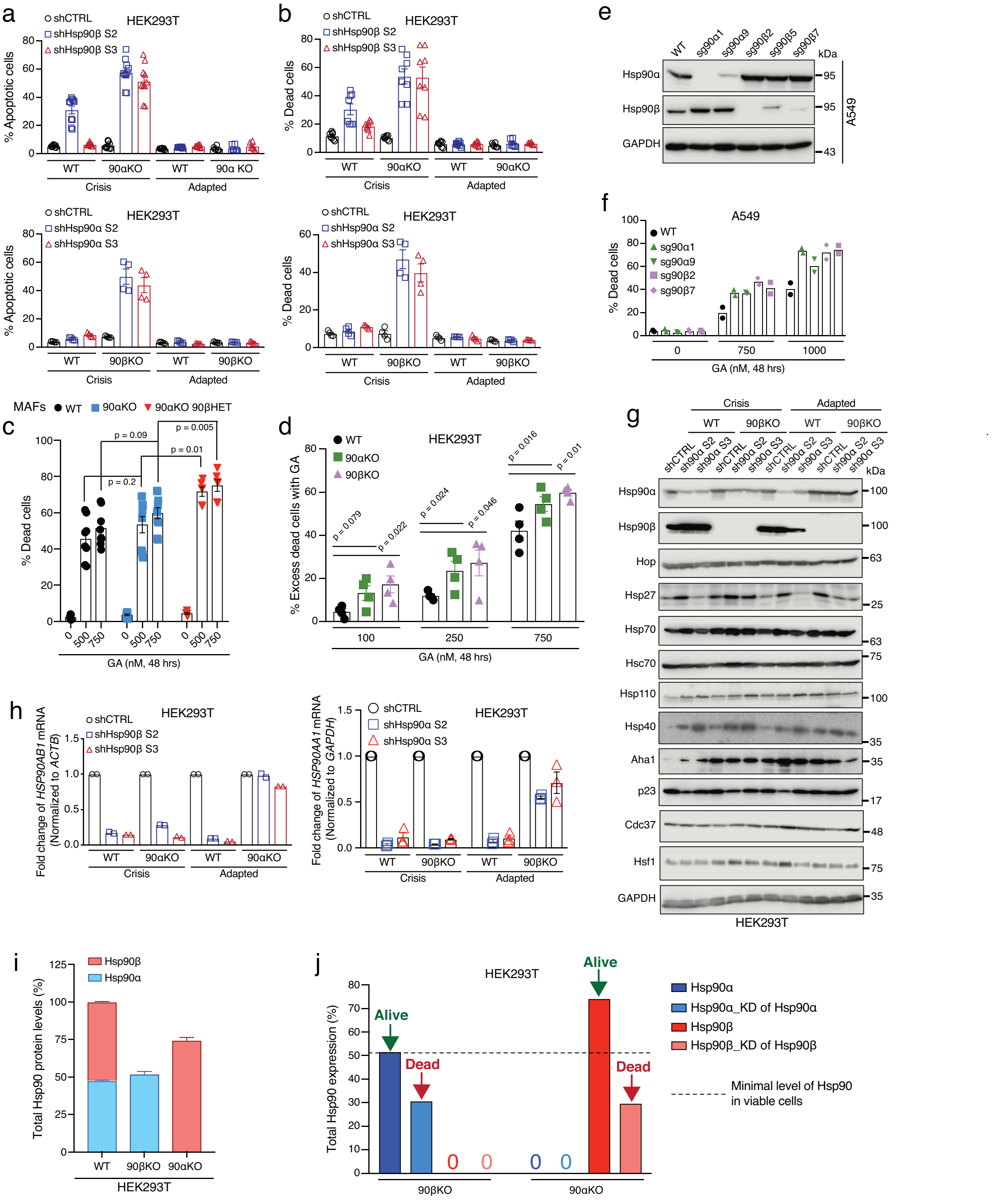

Supplementary Fig. 8 Critical threshold levels of Hsp90 for mammalian life. a-b, Flow cytometric measurements of apoptotic (a) and dead (b) cells upon KD of the remaining Hsp90 isoform in WT and Hsp90α/β KO HEK293T cells during the crisis period (panel a top: n = 10; panel a bottom: n = 4; panel b top: n = 8; panel b bottom: n = 4 biologically independent samples) and after adaptation (panel a top: n = 8 for WT and n = 6 for Hsp90α KO; panel a bottom: n = 4; panel b top: n = 8 for WT and n = 6 for Hsp90α KO; panel b bottom: n = 4 biologically independent samples) (see Fig. 4c for the strategy). **c-d,** Flow cytometric analysis of GA-induced cell death of MAFs

with the indicated genotypes (panel c, n = 8 for WT; n = 10 for 90αKO; n = 6 for 90αKO 90βHET biologically independent samples) and HEK293T (panel d, n = 4 biologically independent samples) cells. For panel d, % excess dead cells = % dead cells in inhibitor-treated sets — % dead cells in the control set. **e,** Immunoblot analysis of Hsp90 mutant clones of A549 cells (designated "sg90α/β"). sg, small guide RNA, used for CRISPR-Cas9-mediated KO/KD (representative of n = 3 biologically independent experiments). **f,** Flow cytometric measurements of GA-induced dead cells of A549 cells as indicated (n = 2 biologically independent samples). **g,** Immunoblots of molecular chaperones, co-chaperones, and stress-related proteins upon KD of Hsp90α in WT and Hsp90β KO HEK293T cells during the crisis period and after adaptation (see Fig. 4c for the strategy; representative of n = 2 biologically independent experiments). GAPDH serves as loading control. **h,** Quantitative measurements of the indicated *HSP90* mRNA by qRT-PCR during the crisis period and after adaptation (*HSP90AB1*, n = 2; *HSP90AA1*, n = 3 biologically independent samples; see Fig. 4c for the strategy). shCTRL values were set to 1 for each genotype and condition. **i,** Total Hsp90 protein levels and relative proportions of the Hsp90α and Hsp90β isoforms in WT and Hsp90α/β KO HEK293T cells measured by quantitative label-free proteomic analysis. Total Hsp90 protein levels in WT cells were set to 100% (n = 3 biologically independent samples). **j,** Graphical representation of the critical threshold of Hsp90 levels of Hsp90α/β KO HEK293T cells (see panel i). KD of the remaining isoform of Hsp90 KO HEK293T cells triggers cell death. Values of Hsp90 levels in KD cells were determined by densitometric measurements (with ImageJ-Fiji) of immunoblot images as shown in Fig. 4e and Supplementary Fig. 8g (n = 2 independent KD experiments). The bar graphs show mean values ± SEM. The statistical significance between the groups was analyzed by two-tailed unpaired Student's t-tests. Source data are provided as a Source Data file.

Supplementary Figure 9

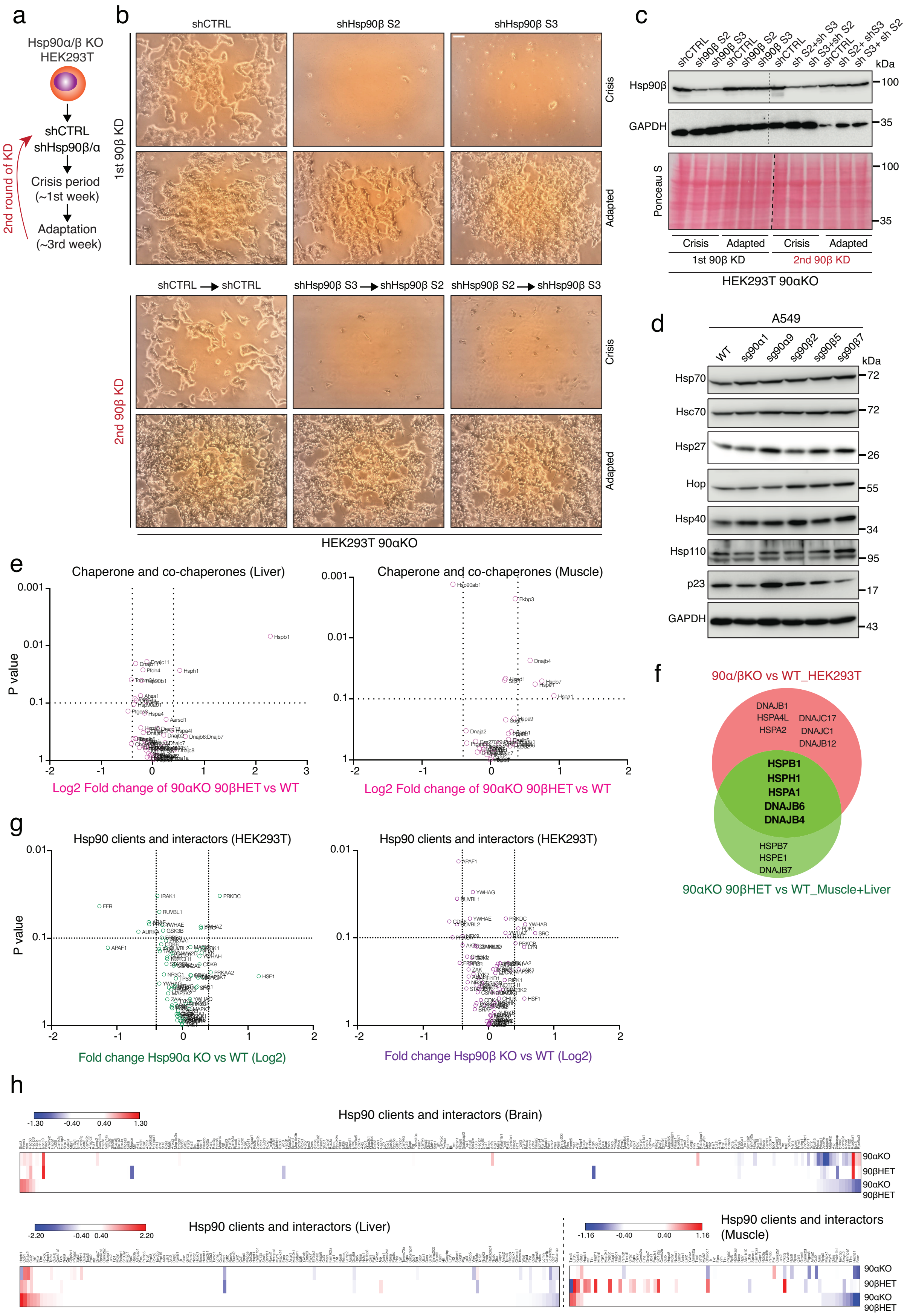

Supplementary Fig. 9 Other molecular chaperones cannot compensate for deficits in Hsp90 levels. a, Strategy for two successive KD of the remaining Hsp90 isoform in Hsp90α/β KO HEK293T cells. **b,** Phase-contrast micrographs of Hsp90α KO HEK293T cells during two successive KD of the mRNA of the remaining *Hsp90β* gene. Note that during the second round of KD, a different targeting shRNA was used to avoid inefficient KD due to target sequence mutation (representative of n = 2 biologically independent experiments). A representative scale bar (100 μm) is shown in the micrograph at the top right. **c,** Immunoblot of Hsp90β from Hsp90α KO HEK293T cells during two successive KD experiments (representative of n = 2 biologically independent experiments). GAPDH and ponceau s-stained nitrocellulose membrane serve as loading controls. **d,** Immunoblots of molecular chaperones, co-chaperones, and stress-related proteins from WT and Hsp90 mutant A549 cells (representative of n = 2 biologically independent experiments). Note that the GAPDH immunoblot is identical to that of Supplementary Fig. 8e since all the data were acquired with the same cell lysates. **e,** Volcano plots of the normalized fold changes of the Hsp70-Hsp90-related chaperones, co-chaperones, and other stress-responsive proteins determined by quantitative label-free proteomic analyses of liver and muscle of the indicated genotypes (n = 2 biologically independent samples). The statistical significance between the groups was analyzed by two-tailed unpaired Student's t-tests. **f,** Venn diagram of cumulated overexpressed (Log2 fold change > 0.4; p value < 0.1) molecular chaperones and co-chaperones in Hsp90 mutant tissues (panel e) and cells (Fig. 4f) compared to respective WT samples (the p-values are included in each corresponding original panel, i.e., panel e and Fig. 4f). **g,** Volcano plots of the normalized fold changes of the Hsp90 clients and interactors determined by quantitative label-free proteomic analysis of Hsp90α/β KO and WT HEK293T cells (n = 3 biologically independent samples). Log2 fold change of > 0.4 or < -0.4 with a p-value of < 0.1 were considered significant differences. Comparisons between samples of different genotypes are shown in different colors. p-values were calculated by a two-tailed unpaired Student's t-tests with Benjamini-Hochberg p-value correction. **h,** Heat maps of the normalized fold changes of the levels of Hsp90 clients and interactors as determined by quantitative label-free proteomic analyses of mouse tissues. Each Hsp90 mutant genotype was compared to the WT counterpart. Red, significantly upregulated; blue, significantly downregulated. Source data are provided as a Source Data file.

Supplementary Figure 10

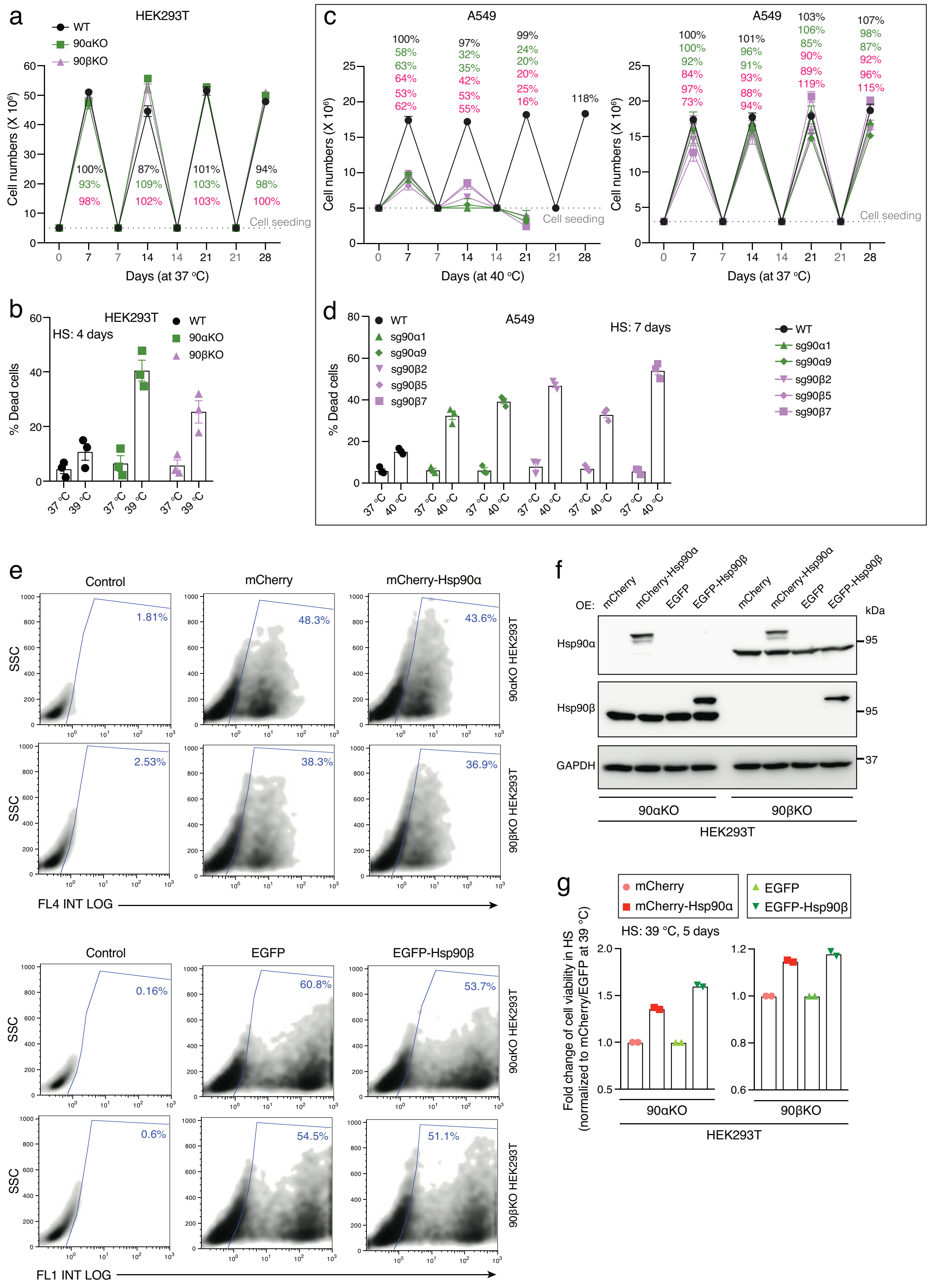

Supplementary Fig. 10 Reduced Hsp90 levels cause lethality upon exposure to long-term mild heat stress. a, Proliferation of WT and Hsp90α/β KO HEK293T cells at 37 °C for the indicated period presented as cell numbers (n = 5 biologically independent experiments). This is a control experiment for Fig. 4g. **b,** Flow cytometric analysis of cell death of WT and Hsp90α/β KO HEK293T cells after 4 days at 37 °C and 39 °C (n = 3 biologically independent experiments). HS, heat shock. **c,** Proliferation of WT and Hsp90α/β mutant A549 cells at 40 °C (left) and 37 °C (right). Cells were reseeded at densities of 3 x 10^6 and 5 x 10^6 every 7th day at 37 °C and 40 °C, respectively (n = 5 biologically independent experiments). **d,** Flow cytometric analysis of cell death of WT and Hsp90α/β mutant A549 cells after 7 days at 37 °C and 40 °C (n = 3 biologically independent experiments). **e,** Flow cytometric evaluation of % transfected cells (blue) 24 hrs after transfection of plasmids expressing mCherry, mCherry-Hsp90α, EGFP, and EGFP-Hsp90β into Hsp90α/β KO HEK293T cells (representative of n = 2 biologically independent experiments). Red fluorescence of mCherry and green fluorescence of EGFP were detected in the FL4 INT LOG and FL1 INT LOG channels, respectively. In each density plot, the positive (fluorescent) cells appear on the right in the area delineated by a blue line. **f,** Immunoblots of endogenous and exogenously overexpressed Hsp90α and Hsp90β of Hsp90α/β KO HEK293T cells (representative of n = 2 biologically independent experiments). Exogenously overexpressed mCherry-Hsp90α and EGFP-Hsp90β fusion proteins have a correspondingly higher molecular weight compared to endogenous Hsp90. **g,** Normalized fold change of cell viability at 39 °C (5 days in culture) of Hsp90α/β KO HEK293T cells expressing mCherry, mCherry-Hsp90α, EGFP, or EGFP-Hsp90β. Cell viability was normalized to 37 °C (n = 2 biologically independent experiments). Values of cells expressing only mCherry or EGFP were set to 1 (black outlined box). Note that the Y-axis starts at 0.5. Bar graphs show mean values ± SEM. Source data are provided as a Source Data file.

Supplementary Figure 11

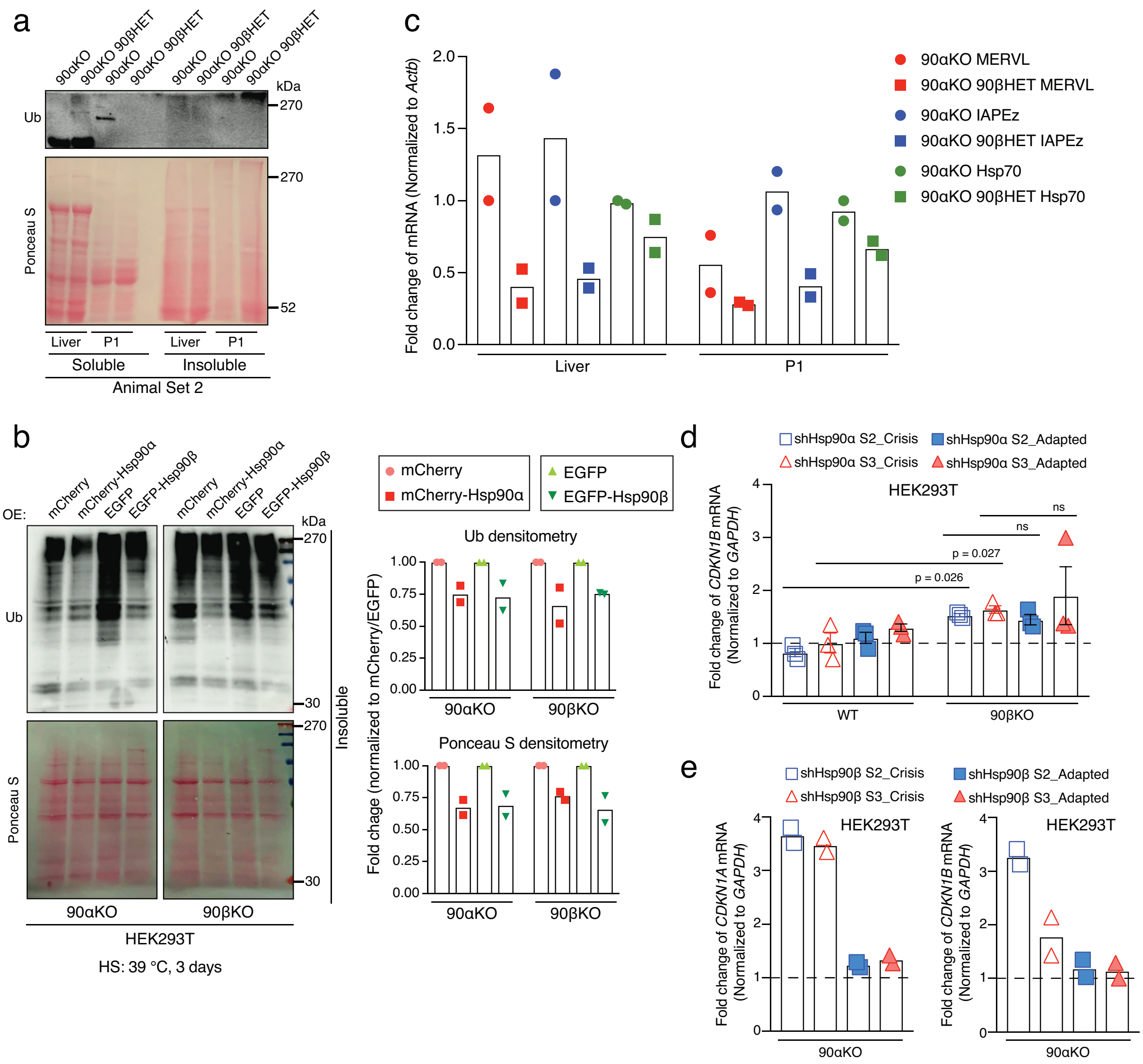

Supplementary Fig. 11 Reduced Hsp90 levels trigger proteotoxicity and accelerated cellular senescence during heat stress adaptation. a, Total (Ponceau S-stained proteins) and polyubiquitinated detergent-soluble and -insoluble proteins as done in Fig. 5b (representative of n = 2 biologically independent experiments). **b,** Total (Ponceau S-stained proteins) and polyubiquitinated detergent-insoluble proteins from Hsp90α/β KO HEK293T cells expressing mCherry, mCherry-Hsp90α, EGFP, or EGFP-Hsp90β. A representative immunoblot is shown on the left, and the relative densitometric quantitation of two biologically independent experiments as bar graphs on the right. Values of cells expressing only mCherry or EGFP were set to 1 (black outlined box). **c,** Quantitative analysis of mRNA transcripts of the cellular retroviral genes MERVL and IAPEz, and of the Hsp70 gene (*Hspa1*) in liver of adult mice and P1 pups of the indicated genotypes (n = 2 independent animal sets). The 90αKO 90βHET P1 pup was stillborn (see Fig. 4a). The values for one experimental set of 90αKO liver was set to 1. **d-e,** mRNA expression of the senescent markers *CDKN1B* (p27) and *CDKN1A* (p21) upon KD of the indicated Hsp90 isoform in WT and Hsp90α/β KO HEK293T cells during the crisis period and after adaptation (panel d, n

= 3 biologically independent samples; panel e, n = 2 biologically independent samples). The values of the shCTRL sets were set to 1 (dashed line). The bar graphs show mean values ± SEM. The statistical significance between the groups was analyzed by two-tailed unpaired Student's t-tests. Source data are provided as a Source Data file.

Supplementary Figure 12

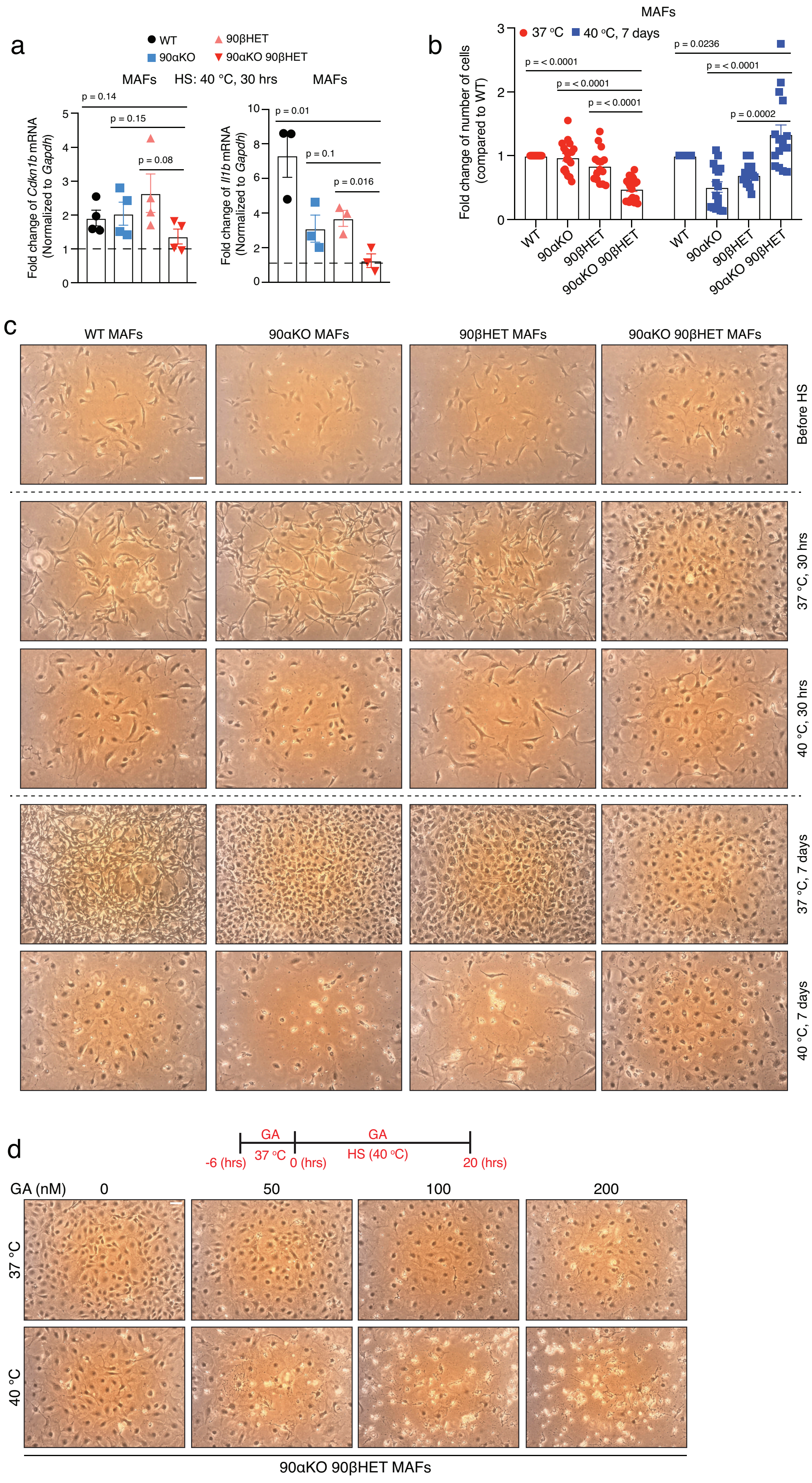

Supplementary Fig. 12 Fine-tuned Hsp90β alleviate chronic heat stress-induced lethality. **a**, HS (40 °C for 30 hrs)-induced mRNA expression of the senescent markers *Cdkn1b* (p27; n = 4 biologically independent samples) and *Il1b* (interleukin 1β; n = 3 biologically independent samples) in MAFs of the indicated genotypes. Expression values at 37 °C were set to 1. **b**, Relative fold change of cell numbers of Hsp90 mutant MAFs at 40 °C (7 days, n = 16 biologically independent samples) and 37 °C (WT, n = 19; 90αKO, n = 18; 90βHET, n = 15; 90αKO 90βHET, n = 19 biologically independent samples) compared to WT (set to 1). **c**, Phase contrast micrographs of WT and Hsp90 mutant MAFs subjected to normal temperature (37 °C) or mild heat shock [40 °C; 30 hrs (representative of n = 3 biologically independent experiments) or 7 days (representative of n = 4 biologically independent experiments)]. **d**, Phase-contrast micrographs of 90αKO 90βHET MAFs exposed to 40 °C (HS) and 37 °C in the absence and presence of GA (representative of n = 2 biologically independent experiments). The flow cytometric measurements of dead cells from the same experiments are shown in Fig. 5g. The experimental scheme is shown above the micrographs. For panels c and d, a representative scale bar (100 μm) is shown in the top left micrographs. The bar graphs show mean values ± SEM. The statistical significance between the groups was analyzed by two-tailed unpaired Student's t-tests. Source data are provided as a Source Data file.

Supplementary Figure 13

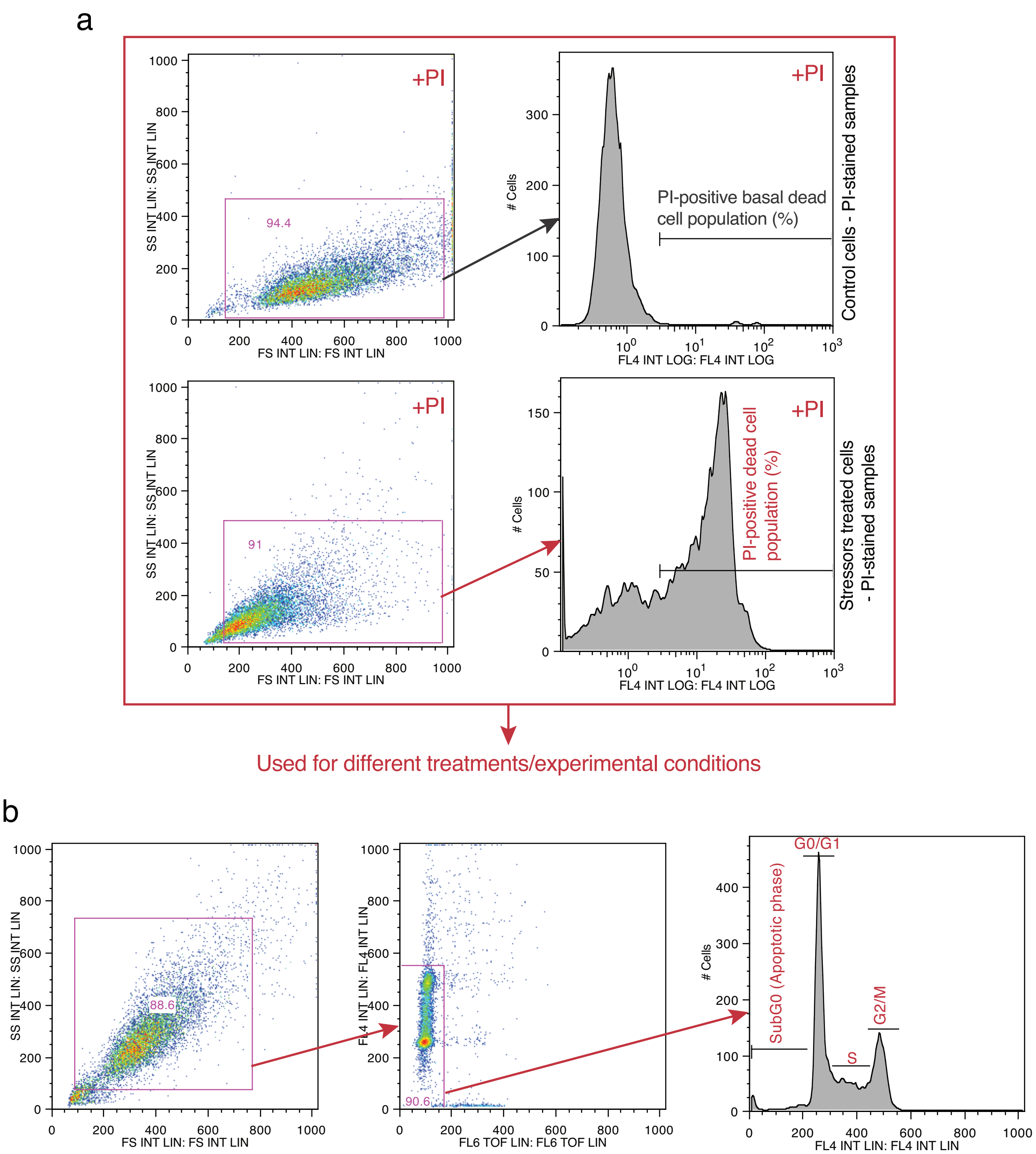

Supplementary Fig. 13 Schematic representation of the FACS gating and analysis strategies. a, FACS gating and analysis strategy for the propidium iodide (PI) positive population of dead cells; related to Fig. 5g, and Supplementary Figs. 8b-d,f, and 10b,d. **b,** FACS gating and analysis strategy of cell cycle experiments; related to Supplementary Fig. 8a.

Supplementary Table 1 The list of oligonucleotides used in this study.

Name of oligonucleotides	Sequences (5´-3´)
Human *HSP90AA1 (HSP90α)* gRNA for CRISPR	GGTTGAGACGTTCGCCTTTC [1]
Human *HSP90AB1 (HSP90β)* gRNA for CRISPR	ATTGCTATTTATTCCTCGTC [1]
Mouse *Hsp90aa1 (HSP90α)* genotyping forward primer	GCTGTTATGGAAGCCTCAGC
Mouse *Hsp90aa1 (HSP90α)* genotyping reverse primer	CACTCCAACCTCCGCAAACTC
Mouse *Hsp90aa1 (HSP90α)* genotyping reverse primer	AGGGTTGTTCTCGGGACTTT
Mouse *Hsp90ab1 (HSP90β)* genotyping forward primer	CCGGCCTATGCAAGTCTGCACAGTGA
Mouse *Hsp90ab1 (HSP90β)* genotyping reverse primer	GTCCATGATGAACACACGGCGGACAT
Mouse *Hsp90ab1 (HSP90β)* genotyping reverse primer	CATGTTCAGTGCATAGCTCACTGACT-CTG
sh-*HSP90AA1* (S2) target sequence	TACTTGGAGGAACGAAGAATA [2]
sh-*HSP90AA1* (S3) target sequence	GTTATCCTACACCTGAAAGAA [2]
sh-*HSP90AB1* (S2) target sequence	CGCATGGAAGAAGTCGATTAG [2]
sh-*HSP90AB1* (S3) target sequence	CTTGTGTTGAAGGCAGTAAAC [2]
sh-Control target sequence	CCTAAGGTTAAGTCGCCCTCG [2]
Human *GAPDH* forward primer for qRT-PCR	GCACAACAGGAAGAGAGAGACC
Human *GAPDH* reverse primer for qRT-PCR	AGGGGAGATTCAGTGTGGTG
Human *HSP90AA1* forward primer for qRT-PCR	CATAACGATGATGAGCAGTACGC
Human *HSP90AA1* reverse primer for qRT-PCR	GACCCATAGGTTCACCTGTGT
Human *HSP90AB1* forward primer for qRT-PCR	GGGTATCGGAAAGCAAGCCT
Human *HSP90AB1* reverse primer for qRT-PCR	ATGAGGGACATGAGTTGGGC
Human *CDKN1A* forward primer for qRT-PCR	AGGTGGACCTGGAGACTCTCAG
Human *CDKN1A* reverse primer for qRT-PCR	TCCTCTTGGAGAAGATCAGCCG
Human *CDKN2A* forward primer for qRT-PCR	CTCGTGCTGATGCTACTGAGGA
Human *CDKN2A* reverse primer for qRT-PCR	GGTCGGCGCAGTTGGGCTCC
Human *CDKN1B* forward primer for qRT-PCR	ATAAGGAAGCGACCTGCAACCG
Human *CDKN1B* reverse primer for qRT-PCR	TTCTTGGGCGTCTGCTCCACAG
Mouse *Gapdh* forward primer for qRT-PCR	AGGTCGGTGTGAACGGATTTG
Mouse *Gapdh* reverse primer for qRT-PCR	GGGGTCGTTGATGGCAACA
Mouse *Hsp90aa1* forward primer for qRT-PCR	GACGCTCTGGATAAAATCCGTT
Mouse *Hsp90aa1* reverse primer for qRT-PCR	TGGGAATGAGATTGATGTGCAG
Mouse *Hsp90ab1* forward primer for qRT-PCR	AAACAAGGAGATTTTCCTCCGC
Mouse *Hsp90ab1* reverse primer for qRT-PCR	CGTCAGGCTCTCATATCGAAT

Mouse *Cdkn1a* forward primer for qRT-PCR	GTCAGGCTGGTCTGCCTCCG
Mouse *Cdkn1a* reverse primer for qRT-PCR	CGGTCCCGTGGACAGTGAGCAG
Mouse *Cdkn2a* forward primer for qRT-PCR	CCCAACGCCCCGAACT
Mouse *Cdkn2a* reverse primer for qRT-PCR	GCAGAAGAGCTGCTACGTGAA
Mouse *Cdkn1b* forward primer for qRT-PCR	AGCAGTGTCCAGGGATGAGGAA
Mouse *Cdkn1b* reverse primer for qRT-PCR	TTCTTGGGCGTCTGCTCCACAG
Mouse *Il1b* forward primer for qRT-PCR	TGGACCTTCCAGGATGAGGACA
Mouse *Il1b* reverse primer for qRT-PCR	GTTCATCTCGGAGCCTGTAGTG
Mouse *MERVL* forward primer for qRT-PCR	TTTCTCAAGGCCCACCAATAGT [3]
Mouse *MERVL* reverse primer for qRT-PCR	GACACCTTTTTTAACTATGCGAGC [3]
Mouse *IAPEz* forward primer for qRT-PCR	GCACCCTCAAAGCCTATCTTA [3]
Mouse *IAPEz* reverse primer for qRT-PCR	TCCCTTGGTCAGTCTGGATTT [3]
Mouse *Ahsa1* forward primer for qRT-PCR	CGCCACCAACGTCAACAAC
Mouse *Ahsa1* reverse primer for qRT-PCR	GGCCAGGAACAGGGTTTTCA
Mouse *Dnajb1* forward primer for qRT-PCR	TTCGACCGCTATGGAGAGGAAG
Mouse *Dnajb1* reverse primer for qRT-PCR	CCGAAGAACTCAGCAAACATGGC
Mouse *Hspa1* forward primer for qRT-PCR	ACAAGTCGGAGAACGTGCAGGA
Mouse *Hspa1* reverse primer for qRT-PCR	GTTGTCCGAGTAGGTGGTGAAG
Mouse *Hspa8* forward primer for qRT-PCR	CCGATGAAGCTGTTGCCTATGG
Mouse *Hspa8* reverse primer for qRT-PCR	CCAAGGGAAAGAGGAGTGACATC
Mouse *Ptges3* forward primer for qRT-PCR	GGAAAGACTGGGAGGATGACTC
Mouse *Ptges3* reverse primer for qRT-PCR	TCATCTGCTCCATCTACTTCTGG
Mouse *Stip1* forward primer for qRT-PCR	TGAGTGCTGGGAACATTGATG
Mouse *Stip1* reverse primer for qRT-PCR	AGTCTCCTTTCTTGGCGTAGG
Mouse *Actb* forward primer for qRT-PCR	GGCTGTATTCCCCTCCATCG
Mouse *Actb* reverse primer for qRT-PCR	CCAGTTGGTAACAATGCCATGT
Firefly luciferase (FFL) forward primer for qRT-PCR	ATTACACCCGAGGGGGATGA
Firefly luciferase (FFL) reverse primer for qRT-PCR	TCGCCTCTCTGATTAACGCC
Renilla luciferase (RLUC) forward primer for qRT-PCR	GGGGTGCTTGTTTGGCATTT
Renilla luciferase (RLUC) reverse primer for qRT-PCR	TCAGGCCATTCATCCCATGAT

Supplementary References

1. Bhattacharya, K. *et al.* The Hsp70-Hsp90 co-chaperone Hop/Stip1 shifts the proteostatic balance from folding towards degradation. *Nat. Commun.* **11**, 5975 (2020).
2. Bennesch, M. A., Segala, G., Wider, D. & Picard, D. LSD1 engages a corepressor complex for the activation of the estrogen receptor α by estrogen and cAMP. *Nucleic Acids Res.* **44**, 8655-8670 (2016).
3. Hummel, B. *et al.* The evolutionary capacitor HSP90 buffers the regulatory effects of mammalian endogenous retroviruses. *Nat. Struct. Mol. Biol.* **24**, 234-242 (2017).
