# Supplementary material for: Translational reprogramming in response to accumulating stressors ensures critical threshold levels of Hsp90 for mammalian life
Source: Nat Commun. 2022 Oct 21;13:6271. doi: 10.1038/s41467-022-33916-3 (PMC9587034; doi:10.1038/s41467-022-33916-3)
Supplement: Supplementary file 3 — Description of Additional Supplementary Files [file 41467_2022_33916_MOESM3_ESM.pdf]

File name: Supplementary Data 1

Description: A subset of the proteomic data of brain, liver, and muscle of WT and Hsp90 mutant mice.

File name: Supplementary Data 2

Description: A subset of the proteomic data of WT and Hsp90α/β KO HEK293T cells.
