# Supplementary material for: Translational reprogramming in response to accumulating stressors ensures critical threshold levels of Hsp90 for mammalian life
Source: Nat Commun. 2022 Oct 21;13:6271. doi: 10.1038/s41467-022-33916-3 (PMC9587034; doi:10.1038/s41467-022-33916-3)
Supplement: Supplementary file 6 — Reporting Summary [file 41467_2022_33916_MOESM6_ESM.pdf]

nature research

Corresponding author(s): Didier Picard

Last updated by author(s): Sep 19, 2022

Reporting Summary

Nature Research wishes to improve the reproducibility of the work that we publish. This form provides structure for consistency and transparency in reporting. For further information on Nature Research policies, see our Editorial Policies and the Editorial Policy Checklist.

Statistics

For all statistical analyses, confirm that the following items are present in the figure legend, table legend, main text, or Methods section.

n/a	Confirmed	
☐	☒	The exact sample size (*n*) for each experimental group/condition, given as a discrete number and unit of measurement
☐	☒	A statement on whether measurements were taken from distinct samples or whether the same sample was measured repeatedly
☐	☒	The statistical test(s) used AND whether they are one- or two-sided<br>*Only common tests should be described solely by name; describe more complex techniques in the Methods section.*
☒	☐	A description of all covariates tested
☒	☐	A description of any assumptions or corrections, such as tests of normality and adjustment for multiple comparisons
☐	☒	A full description of the statistical parameters including central tendency (e.g. means) or other basic estimates (e.g. regression coefficient) AND variation (e.g. standard deviation) or associated estimates of uncertainty (e.g. confidence intervals)
☐	☒	For null hypothesis testing, the test statistic (e.g. *F*, *t*, *r*) with confidence intervals, effect sizes, degrees of freedom and *P* value noted<br>*Give P values as exact values whenever suitable.*
☒	☐	For Bayesian analysis, information on the choice of priors and Markov chain Monte Carlo settings
☒	☐	For hierarchical and complex designs, identification of the appropriate level for tests and full reporting of outcomes
☐	☒	Estimates of effect sizes (e.g. Cohen's *d*, Pearson's *r*), indicating how they were calculated

Our web collection on statistics for biologists contains articles on many of the points above.

Software and code

Policy information about availability of computer code

Data collection	Mass spectrometry data acquisition was performed with Xcalibur 4.2 (Thermo Fisher).<br>FACS data were collected by using a FACS Gallios (Beckman Coulter) flowcytometer.<br>Quantitative RT PCR data were collected by using a Bio-Rad CFX96/Connect qPCR Instrument.<br>Polysome profiling data were collected with TracerDAQ Pro data acquisition software (2.3.4.0)
Data analysis	FlowJo 8.7 (https://www.flowjo.com/)<br>MaxQuant 1.6.14.0 and 1.6.3.4, Jürgen Cox Lab, Max Planck Institute of Biochemistry (https://maxquant.org/)<br>ImageJ-Fiji 2.0.0<br>DinoXcope 2.0.2<br>Graph pad prism 8.0<br><br>All other details are provided in the Methods sections.

For manuscripts utilizing custom algorithms or software that are central to the research but not yet described in published literature, software must be made available to editors and reviewers. We strongly encourage code deposition in a community repository (e.g. GitHub). See the Nature Research guidelines for submitting code & software for further information.

Data

Policy information about availability of data

All manuscripts must include a data availability statement. This statement should provide the following information, where applicable:

- Accession codes, unique identifiers, or web links for publicly available datasets
- A list of figures that have associated raw data
- A description of any restrictions on data availability

The mass spectrometry proteomics data have been deposited to the ProteomeXchange Consortium via the PRIDE partner repository with the dataset identifier PXD031456 and for a subset in Supplementary Data 1-2. Source data of uncropped immunoblot images and the individual data points are provided with this paper.

The UniProt mouse reference proteome (RefProt) database of November 2019 (55'431 sequences) and human RefProt database of October 2017 (71'803 sequences) were used, supplemented with sequences of common contaminants.

Hsp90Int.DB https://www.picard.ch/Hsp90Int/index.php

Field-specific reporting

Please select the one below that is the best fit for your research. If you are not sure, read the appropriate sections before making your selection.

☒ Life sciences ☐ Behavioural & social sciences ☐ Ecological, evolutionary & environmental sciences

For a reference copy of the document with all sections, see nature.com/documents/nr-reporting-summary-flat.pdf

Life sciences study design

All studies must disclose on these points even when the disclosure is negative.

Sample size	No sample size was predetermined. Based on the consistency of quantitative differences between groups and replicates, sample size were determined to be adequate in a particular experimental setting. Therefore, Three and more independent results were used to perform statistical analyses unless explained otherwise in the manuscript.
Data exclusions	No data were excluded from analysis.
Replication	All the experiments in the article were reliably reproduced. All the attempts for the data replication were successful. Information about the number of replicates is provided in the figure legends.
Randomization	We did not perform any formal randomization techniques for in vitro experiments and this was not required for our study. Samples were distributed based on the genotypes/treatments/experimental conditions applied and their identity was known during experimentation and data analysis. Sex and age matched animals of the designated genotypes were randomly picked for the experiments without any bias. Human research participants were not involved in this study.
Blinding	Investigators were not blinded to group allocation during data collection and/or analysis. The results of the experiments provided quantitative data that were analyzed with the appropriate statistical tests to evaluate differences and statistical significance, so blinding was not relevant.

Reporting for specific materials, systems and methods

We require information from authors about some types of materials, experimental systems and methods used in many studies. Here, indicate whether each material, system or method listed is relevant to your study. If you are not sure if a list item applies to your research, read the appropriate section before selecting a response.

Materials & experimental systems

n/a	Involved in the study	
☐	☒	Antibodies
☐	☒	Eukaryotic cell lines
☒	☐	Palaeontology and archaeology
☐	☒	Animals and other organisms
☒	☐	Human research participants
☒	☐	Clinical data
☒	☐	Dual use research of concern

Methods

n/a	Involved in the study	
☒	☐	ChIP-seq
☐	☒	Flow cytometry
☒	☐	MRI-based neuroimaging

Antibodies

Antibodies used

Mouse monoclonal anti-Hop (DS14F5) Enzo Life Sciences ADI-SRA-1500
Mouse monoclonal anti-GAPDH (6C5) HyTest Ltd. 5G4
Mouse monoclonal anti-Hsp70 [C92F3A-5] StressMarq SMC-100
Mouse monoclonal anti-Hsc70 [1F2-H5] StressMarq SMC-151
Rabbit polyclonal anti-Raf1 Santa Cruz Biotechnology Sc-133
Mouse monoclonal anti-beta-Actin (8H10D10) Cell Signaling Technology 3700
Rabbit monoclonal anti-Ub (Apu2) Sigma/Millipore 05-1307
Rat monoclonal anti-Hsp90Alpha (9D2) Enzo Lifesciences ADI-SPA-840
Rabbit polyclonal anti-Hsp90Alpha Synaptic Systems 380003
Mouse monoclonal anti-Hsp90Beta (H90-10) GENEVA ANTIBODY FACILITY ABCD_AO870
Mouse monoclonal anti-p23 (JJ3) Toft Lab, Mayo Clinic/Gift, ThermoFisher/MA3-414
Rabbit monoclonal anti-Cdc37 (D11A3) Cell Signaling Technology 4793
Mouse monoclonal anti-Akt (40D4) Cell Signaling Technology 2920
Rabbit monoclonal anti-mTOR (7C10) Cell Signaling Technology 2983
Rabbit polyclonal anti-Phospho-mTOR (Ser2448) Cell Signaling Technology 2971
Rabbit monoclonal anti-Phospho-S6 Ribosomal Protein (Ser235/236) (D57.2.2E) Cell Signaling Technology 4858
Rabbit polyclonal anti-eIF2α Cell Signaling Technology 9722
Rabbit monoclonal anti-Phospho-eIF2α (Ser51) (119A11) Cell Signaling Technology 3597
Rabbit polyclonal anti-Hsf1 Enzo Lifesciences ADI-SPA-901
Rabbit polyclonal anti-Hsp40/Hdj1 Enzo Lifesciences ADI-SPA-400
Rabbit polyclonal anti-Hsp110 Enzo Lifesciences ADI-SPA-1101
Rat monoclonal anti-Aha1 [25F2.D9] StressMarq SMC-172
Mouse monoclonal anti-Hsp25/27 [8A7] StressMarq SMC-114
Mouse monoclonal anti-Puromycin (12D10) Sigma/Millipore MABE343
Anti-rat IgG-HRP Invitrogen 629520
Anti-mouse IgG-HRP Invitrogen 31430
Anti-rabbit IgG-HRP Invitrogen 31460

Validation

Mouse monoclonal anti-Hop Enzo Life Sciences ADI-SRA-1500: Knockout validated in Nat Commun 11, Article number: 5975 (2020). Detects mouse and human antigen, western blot (WB) compatible.
Mouse monoclonal anti-GAPDH HyTest Ltd. 5G4: Hybridoma clones have been derived from hybridization of Sp2/0 myeloma cells with spleen cells of Balb/c mice immunized with human or rabbit GAPDH. Detects mouse and human antigen, WB compatible.
Mouse monoclonal anti-Hsp70 StressMarq SMC-100: Validation has been done by WB using several human cell lysates. Detects mouse and human antigen, WB compatible.
Mouse monoclonal anti-Hsc70 StressMarq SMC-151: Validation has been done by WB using several human cell lysates and the purified protein. Detects mouse and human antigen, WB compatible.
Rabbit polyclonal anti-Raf1 Santa Cruz Biotechnology Sc-133: Epitope mapping at the C-terminus of Raf-1 of human origin. Köhler, M. et al. 2016. The EMBO journal. 35: 143-61. Detects mouse and human antigen, WB compatible. Discontinued.
Mouse monoclonal anti-beta-Actin Cell Signaling Technology 3700: Validation has been done by WB using several cell lysates. Detects mouse and human antigen, WB compatible.
Rabbit monoclonal anti-Ub Sigma/Millipore 05-1307: Validation has been done by WB using antibody-K48 linked multiubiquitin chain immunocomplex. Detects mouse and human antigen, WB compatible. Discontinued.
Rat monoclonal anti-Hsp90Alpha (9D2) Enzo Lifesciences ADI-SPA-840: This antibody was validated by immunoblot analysis using purified human Hsp90alpha recombinant protein along with human Hsp90beta recombinant protein as a negative control. Knockout validated in this paper. Detects human antigen, WB and IP compatible.
Rabbit polyclonal anti-Hsp90Alpha Synaptic Systems 380003: Knockdown and knockout validated in this paper. Detects mouse and human antigen, WB compatible.
Mouse monoclonal anti-Hsp90Beta (H90-10) GENEVA ANTIBODY FACILITY ABCD_AO870: This antibody was validated by immunoblot analysis using purified human Hsp90beta recombinant protein along with human Hsp90alpha recombinant protein as a negative control. Also this antibody is knockout validated in this paper. Detects mouse and human antigen, WB and immunoprecipitation compatible.
Mouse monoclonal anti-p23 (JJ3) Toft Lab, Mayo Clinic/Gift, ThermoFisher/MA3-414: This antibody was validated by immunoblot analysis using several mouse and human cell extracts. Detects mouse and human antigen, WB compatible.
Rabbit monoclonal anti-Cdc37 Cell Signaling Technology 4793: Validation has been done by WB using several human cell lysates. Detects mouse and human antigen, WB compatible.
Mouse monoclonal anti-Akt Cell Signaling Technology 2920: Validation has been done by WB using several mouse and human cell lysates and also using purified recombinant proteins as antigen. Detects mouse and human antigen, WB compatible.
Rabbit monoclonal anti-mTOR Cell Signaling Technology 2983: Validation has been done by WB using several mouse and human cell lysates and knockdown validated. Detects mouse and human antigen, WB compatible.
Rabbit polyclonal anti-Phospho-mTOR (Ser2448) Cell Signaling Technology 2971: Validation has been done by WB using serum starved and EGF treated cell lysates. Detects mouse and human antigen, WB compatible.
Rabbit monoclonal anti-Phospho-S6 Ribosomal Protein (Ser235/236) Cell Signaling Technology 4858: Validation has been done by WB using growth factor treated cell lysates. Detects mouse and human antigen, WB compatible.
Rabbit polyclonal anti-eIF2α Cell Signaling Technology 9722: Validation has been done by WB using PC12 cell lysates. Detects mouse and human antigen, WB compatible.
Rabbit monoclonal anti-Phospho-eIF2α (Ser51) Cell Signaling Technology 3597: Validation has been done by WB using thapsigargin treated cell lysates. Detects mouse and human antigen, WB compatible.
Rabbit polyclonal anti-Hsf1 Enzo Lifesciences ADI-SPA-901: Recombinant human HSF1 was used as immunogen. Validation has been done by WB using heat shocked and non-heat shocked cell lysates. Detects mouse and human antigen, WB compatible.
Rabbit polyclonal anti-Hsp40/Hdj1 Enzo Lifesciences ADI-SPA-400: Validation has been done by WB using heat shocked and non-heat shocked cell lysates and also with purified recombinant protein. Detects mouse and human antigen, WB compatible.
Rabbit polyclonal anti-Hsp110 Enzo Lifesciences ADI-SPA-1101: Synthetic peptide corresponding to a portion of hamster HSP110 was

used as immunogen. The sequence is completely conserved in human and mouse. Detects mouse and human antigen, WB compatible.
Rat monoclonal anti-Aha1 StressMarq SMC-172: Recombinant full length mouse Aha1 protein was used as antigen. Validation has been done by WB using several human cell lysates. Detects mouse and human antigen, WB compatible.
Mouse monoclonal anti-Hsp25/27 StressMarq SMC-114: Human Hsp27 peptide was used as antigen. Validation has been done by WB using rat lung lysates. Detects mouse and human antigen, WB compatible.
Mouse monoclonal anti-Puromycin Sigma/Millipore MABE343: Anti-Puromycin antibody detects puromycin incorporated protein and can be inhibited by cycloheximide. WB compatible.

Eukaryotic cell lines

Policy information about cell lines

Cell line source(s)	HEK293T, A549, and RPE1 cell lines were purchased from the American Type Culture Collection (ATCC). MAFs were established in the laboratory as explained in the Methods section.
Authentication	Cell lines were not authenticated by ourselves.
Mycoplasma contamination	All cell lines were tested to be mycoplasma negative.
Commonly misidentified lines (See ICLAC register)	None of the cell lines used in this study were found in the commonly misidentified cell lines database.

Animals and other organisms

Policy information about studies involving animals; ARRIVE guidelines recommended for reporting animal research

Laboratory animals	Gene knockout mice and ROSA26::FLPe deleter strain of C57BL/6N strain background, both male and female animals of 3-9 months of age are included into the study. Details of the mouse lines and breeding strategies are mentioned in the Methods section.
Wild animals	No wild animals were used in this study.
Field-collected samples	No field-collected samples were used in this study.
Ethics oversight	Animal breeding and all animal experiments were carried out according to Swiss laws and with formal authorizations from the State (Direction générale de la santé, République et Canton de Genève) and Federal (Office fédéral de la sécurité alimentaire et des affaires vétérinaires) authorities.

Note that full information on the approval of the study protocol must also be provided in the manuscript.

Flow Cytometry

Plots

Confirm that:

☒ The axis labels state the marker and fluorochrome used (e.g. CD4-FITC).

☒ The axis scales are clearly visible. Include numbers along axes only for bottom left plot of group (a 'group' is an analysis of identical markers).

☒ All plots are contour plots with outliers or pseudocolor plots.

☒ A numerical value for number of cells or percentage (with statistics) is provided.

Methodology

Sample preparation	Described in detail in Methods section for each experiment.
Instrument	FACS Gallios from Beckman Coulter.
Software	Data acquisition was done by FACS Gallios specific acquisition software. All data analyses were performed with FlowJo.
Cell population abundance	We only used the flow cytometry to analyze the cells, not to sort them. For all flow cytometric experiments we acquired/analyzed at least 10,000 cells per sample.
Gating strategy	Preliminary cell population was gated from FSC/SSC plot for all the flow cytometry experiments. Boundaries of the positive and negative populations were determined by using unstained cells. For the cell cycle analyses, different cell cycle phases were determined by the population density and population distribution.

☒ Tick this box to confirm that a figure exemplifying the gating strategy is provided in the Supplementary Information.
