# Supplementary material for: Translational reprogramming in response to accumulating stressors ensures critical threshold levels of Hsp90 for mammalian life
Source: Nat Commun. 2022 Oct 21;13:6271. doi: 10.1038/s41467-022-33916-3 (PMC9587034; doi:10.1038/s41467-022-33916-3)

Figure 2

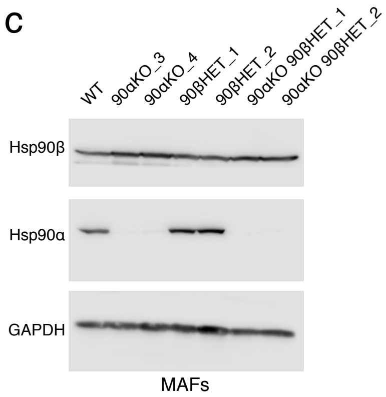
c
WT
90αKO_3
90αKO_4
90βHET_1
90βHET_2
90αKO 90βHET_1
90αKO 90βHET_2
Hsp90β
Hsp90α
GAPDH
MAFs

Figure 3

a

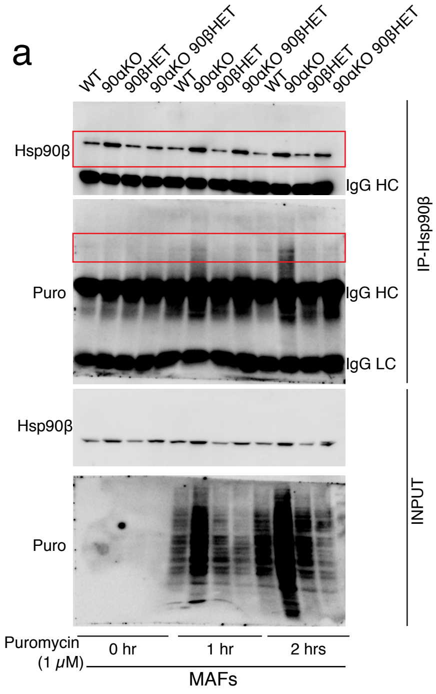

WT
90αKO
90βHET
90αKO 90βHET
WT
90αKO
90βHET
90αKO 90βHET
WT
90αKO
90βHET
90αKO 90βHET
Hsp90β
IgG HC
Puro
IgG HC
IgG LC
IP-Hsp90β
Hsp90β
Puro
INPUT
Puromycin
(1 μM)
0 hr
1 hr
2 hrs
MAFs

g

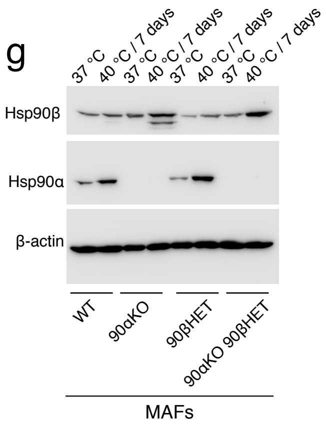

37 °C
40 °C / 7 days
37 °C
40 °C / 7 days
37 °C
40 °C / 7 days
37 °C
40 °C / 7 days
Hsp90β
Hsp90α
β-actin
WT
90αKO
90βHET
90αKO 90βHET
MAFs

Figure 4

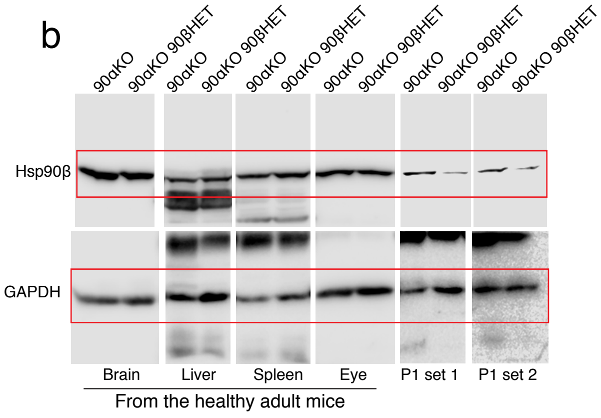
b
90αKO
90αKO 90βHET
Hsp90β
GAPDH
Brain
Liver
Spleen
Eye
P1 set 1
P1 set 2
From the healthy adult mice

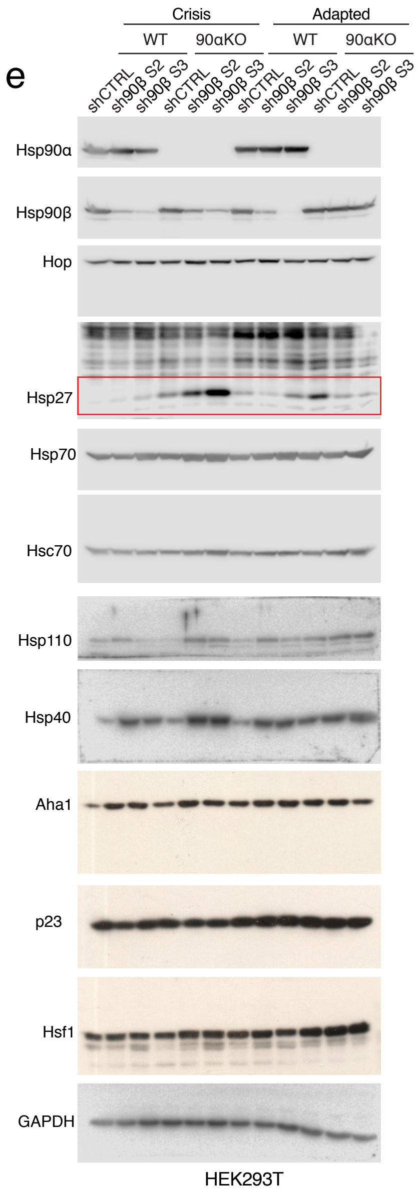
e
Crisis
Adapted
WT
90αKO
shCTRL
sh90β S2
sh90β S3
Hsp90α
Hsp90β
Hop
Hsp27
Hsp70
Hsc70
Hsp110
Hsp40
Aha1
p23
Hsf1
GAPDH
HEK293T

Figure 5

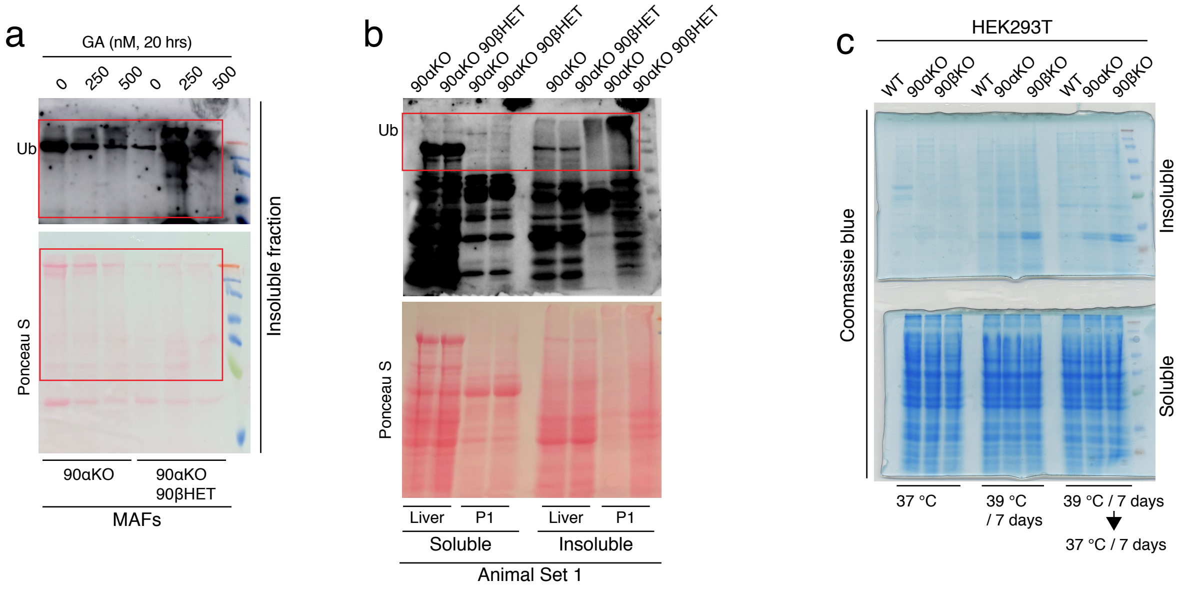

a
GA (nM, 20 hrs)
0
250
500
0
250
500
Ub
Ponceau S
Insoluble fraction
90αKO
90αKO 90βHET
MAFs
b
90αKO
90αKO 90βHET
90αKO
90αKO 90βHET
90αKO
90αKO 90βHET
90αKO
90αKO 90βHET
Ub
Ponceau S
Liver
P1
Liver
P1
Soluble
Insoluble
Animal Set 1
c
HEK293T
WT
90αKO
90βKO
WT
90αKO
90βKO
WT
90αKO
90βKO
Coomassie blue
Insoluble
Soluble
37 °C
39 °C / 7 days
39 °C / 7 days
37 °C / 7 days

Supplementary Figure 2

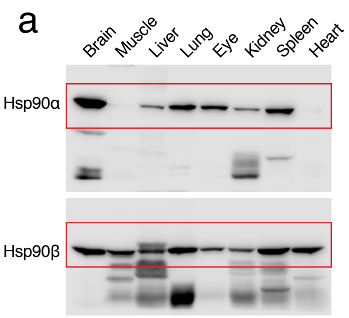

Supplementary Figure 4

a

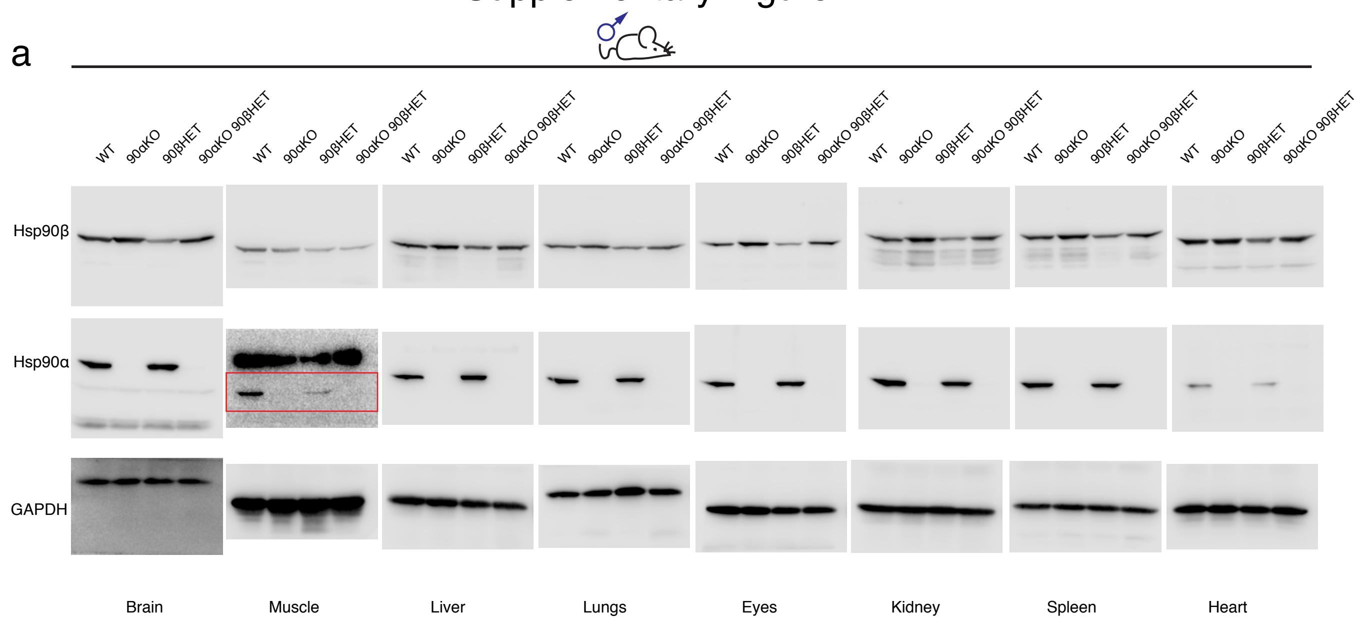

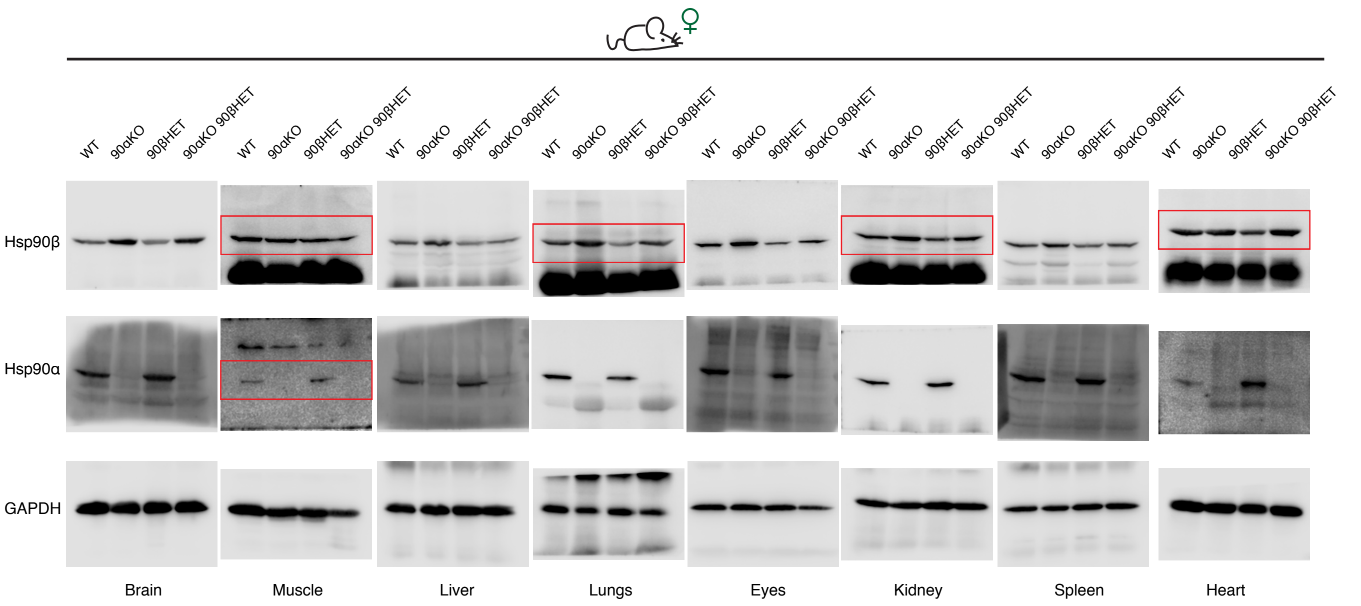

c

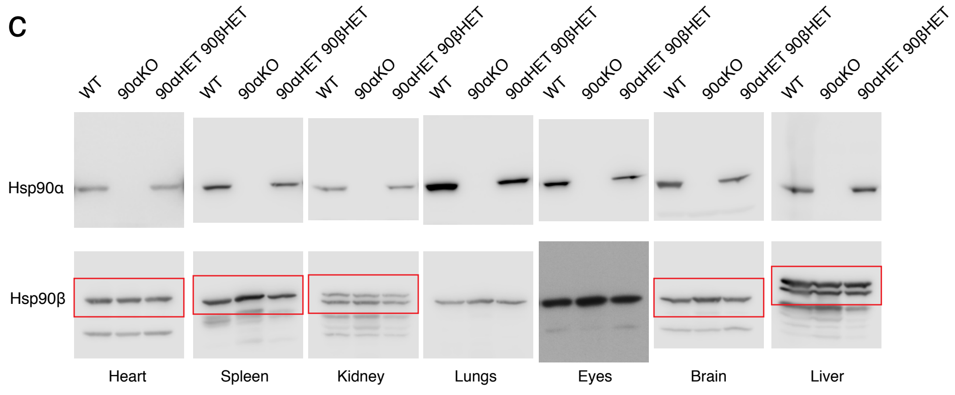

Supplementary Figure 6

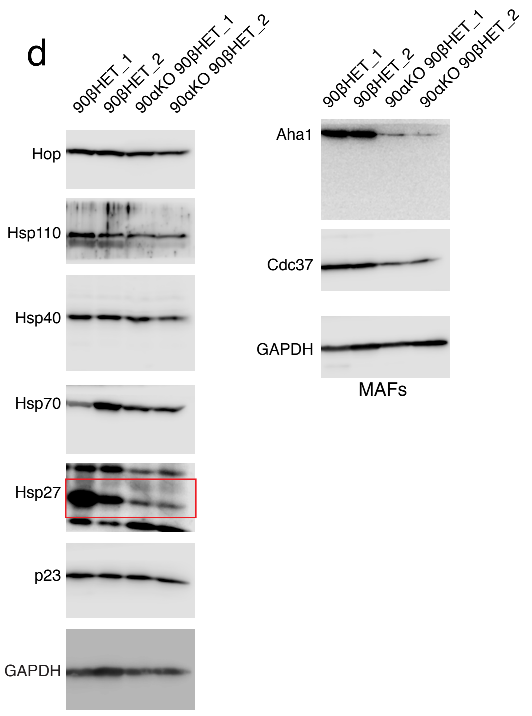

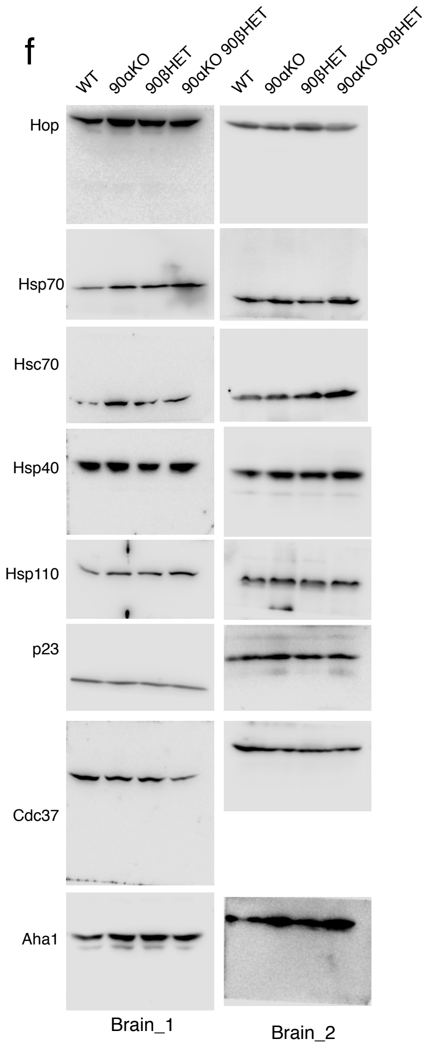

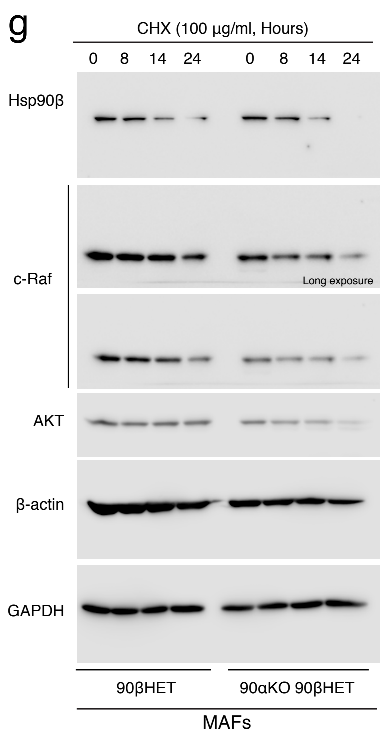

Supplementary Figure 7

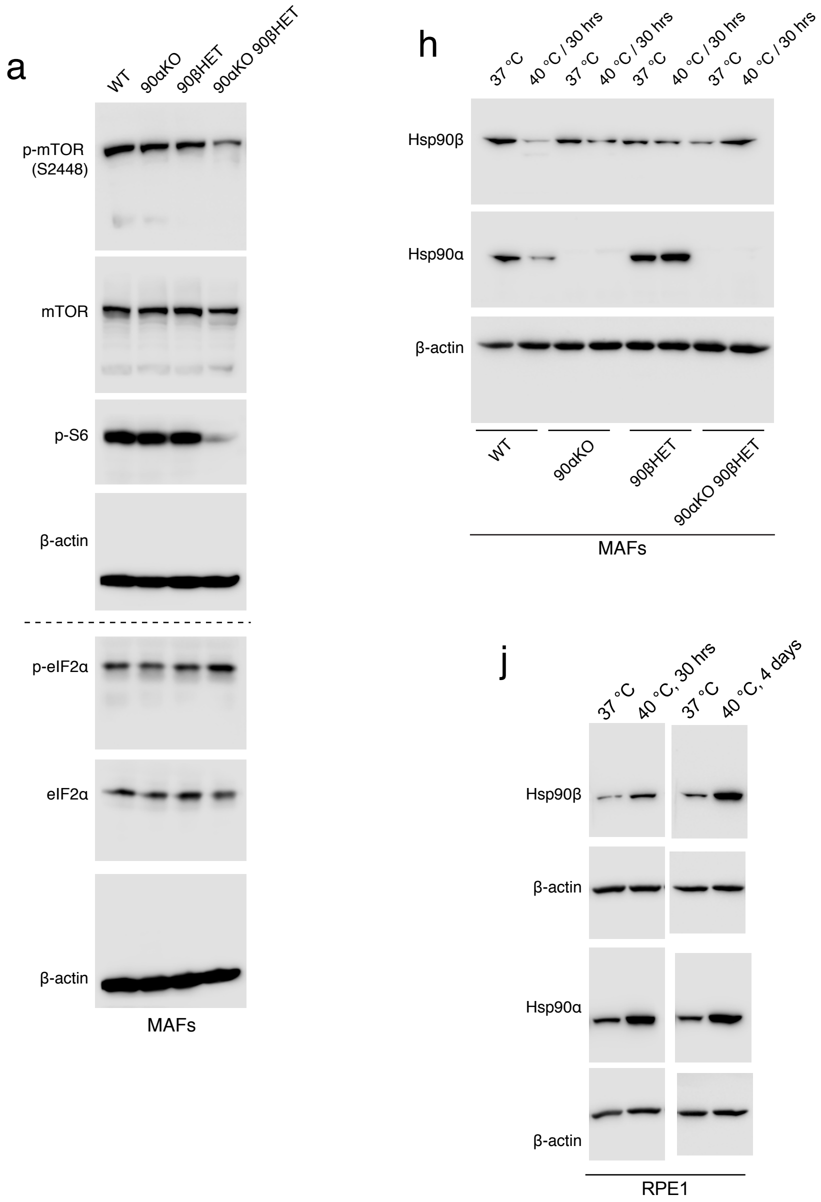

Supplementary Figure 8

e

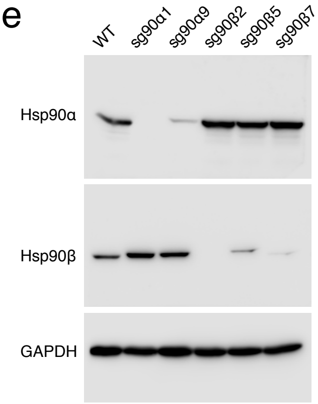

g

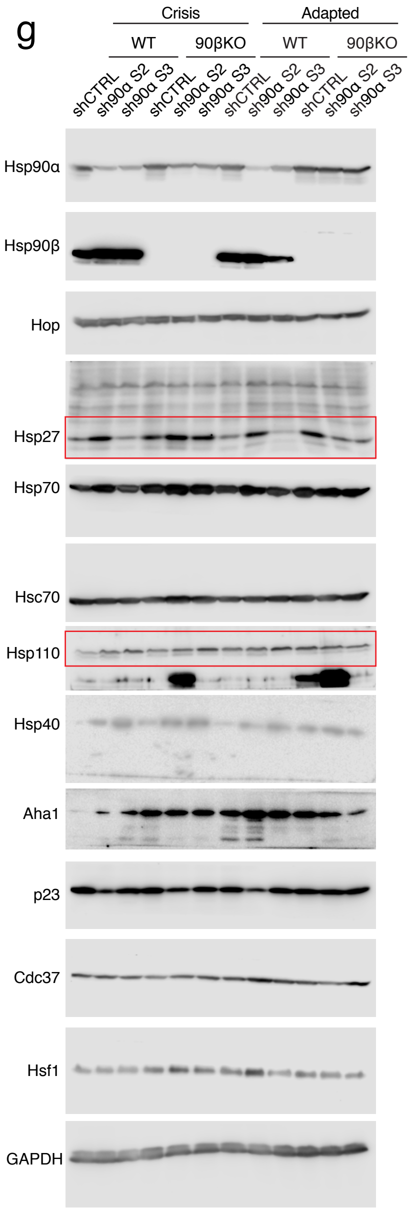

Supplementary Figure 9

c

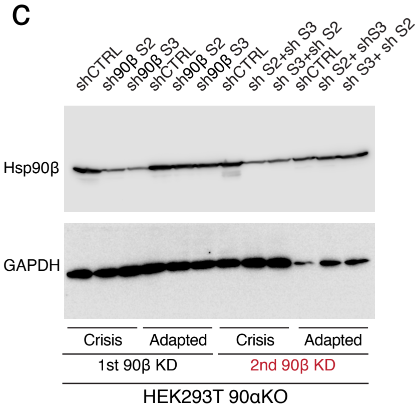

d

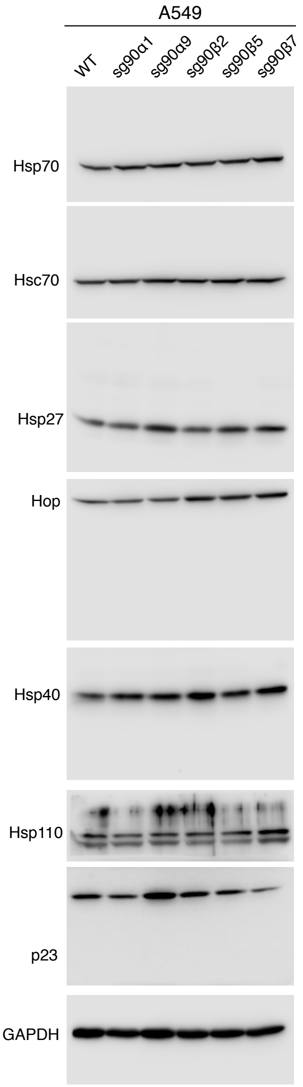

Supplementary Figure 10

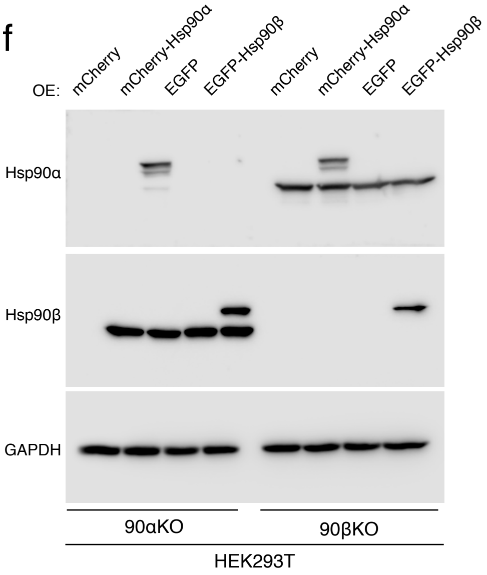

Supplementary Figure 11

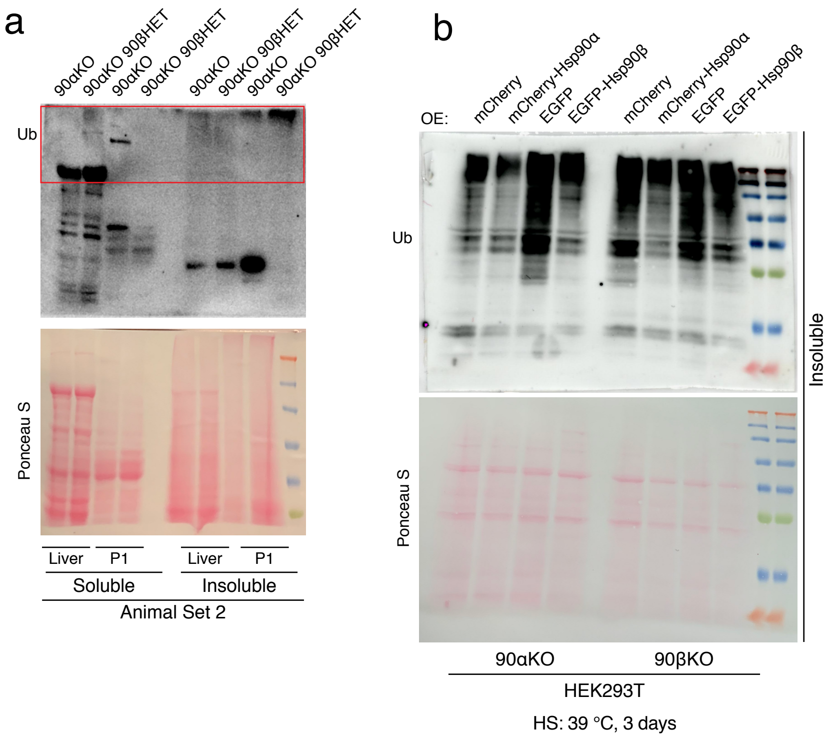

Supplement: Supplementary file 7 — Source Data [file 41467_2022_33916_MOESM7_ESM.zip › Source data file 1.pdf]
